# Muscle–Adipose Aging Dynamics and Inflammaging Patterns Across Ethnically Diverse Populations: Exploring Potential Roles of Lipolysis

**DOI:** 10.1002/jcsm.70316

**Published:** 2026-06-04

**Authors:** Fei‐Yuan Hsiao, Jamie Chi‐Yun Wu, I‐Tzu Chen, Yu‐Ting Lin, Lin‐Chieh Meng, Liang‐Kung Chen

**Affiliations:** ^1^ Graduate Institute of Clinical Pharmacy, College of Medicine National Taiwan University Taipei Taiwan; ^2^ School of Pharmacy, College of Medicine National Taiwan University Taipei Taiwan; ^3^ Department of Pharmacy National Taiwan University Hospital Taipei Taiwan; ^4^ Department of Biostatistics, Epidemiology and Informatics, Perelman School of Medicine University of Pennsylvania Philadelphia Pennsylvania USA; ^5^ Center for Geriatrics and Gerontology Veterans General Hospital Taipei Taiwan; ^6^ Center for Healthy Longevity and Aging Sciences National Yang Ming Chiao Tung University Taipei Taiwan; ^7^ Taipei Municipal Gan‐Dau Hospital (Managed by Taipei Veterans General Hospital) Taipei Taiwan

## Abstract

**Background:**

Muscle–adipose aging reflects interactions between body composition changes and chronic inflammatory processes, but age‐related patterns in these measures across ethnically diverse populations remain incompletely described. Understanding population‐specific patterns is crucial for developing tailored prevention and intervention strategies. We aimed to characterize age‐ and sex‐specific patterns of muscle‐related measures, adiposity, inflammatory markers and lipolysis‐related biomarkers within large cohorts from the United Kingdom, the United States and Taiwan, and to examine how these observed patterns varied across cohorts.

**Methods:**

In this cross‐cohort comparative study, we analysed data from UK Biobank (*n* = 35 436), US NHANES (*n* = 9157) and Taiwan NAHSIT (*n* = 4959). We examined age‐related trajectories of muscle mass, adiposity, muscle‐to‐fat ratio, and biomarkers of inflammation and lipolysis. Inflammaging was assessed using C‐reactive protein (CRP) and insulin‐like growth factor‐1 (IGF‐1) decline profiles. Lipolytic activity was evaluated using circulating ketone bodies (β‐hydroxybutyrate, acetoacetate, acetone) in the UK cohort. Age‐specific patterns were described across populations, with particular attention to masking effects of body mass index (BMI) adjustment and late‐life metabolic selection.

**Results:**

Muscle–adipose aging patterns differed substantially across populations. Taiwanese men, despite having lower absolute appendicular lean mass, showed steeper cross‐sectional differences while maintaining higher muscle‐to‐fat ratios than other cohorts. Inflammaging patterns were consistent across cohorts, with CRP increasing by up to 90% and IGF‐1 declining by 20.3%–23.8% from ages 45 to 79, consistent with a potential link between chronic inflammation and anabolic resistance. BMI‐adjusted muscle mass showed greater deterioration with age compared with height‐adjusted measures, suggesting conventional assessments may mask sarcopenic progression. Adiposity trajectories peaked earlier in Western populations (55–59 years) than in Taiwanese adults (60–64 years), followed by late‐life declines of 10.3%–23.5%, indicative of differential lipolytic activation. In the UK cohort, ketone body concentrations increased with age, suggesting higher fat oxidation in older age groups. Among adults aged 80 years or older, reversals in the muscle‐to‐fat ratio suggested metabolic selection effects across all populations.

**Conclusions:**

Muscle–adipose aging reflects population‐specific patterns of inflammatory and lipolytic reprogramming, with metabolic efficiency—rather than absolute tissue quantity—emerging as a key determinant of healthy aging. Establishing ethnically tailored reference standards may improve the precision of aging‐related risk stratification and interventions across diverse populations.

## 1 Introduction

Aging represents a complex, multisystem physiological process encompassing numerous progressive changes that substantially influence morbidity, mortality and quality of life. Among these diverse age‐related alterations, body composition changes—including the decline in skeletal muscle mass and function, accumulation of adipose tissue and associated dysregulation of metabolic homeostasis—constitute characteristic features of the aging phenotype [1]. Importantly, the magnitude and pattern of these body composition changes exhibit substantial inter‐ethnic variability across racial and ethnic groups [2]. Elucidating these intricate biological interactions and ethnic‐specific variations across geographically diverse populations is imperative for the development of evidence‐based, population‐specific interventions to optimize healthspan and promote healthy longevity on a global scale [3].

Beyond its mechanical functions, skeletal muscle has been increasingly recognized as a vital endocrine organ that communicates with various tissues through myokines and complex signalling pathways [4]. The muscle‐organ crosstalk has demonstrated significant modulatory effects on metabolic, bone, cardiovascular and brain health [5, 6]. Concurrently, adipose tissue serves as an endocrine organ, releasing adipokines that regulate inflammation, insulin sensitivity and energy homeostasis [7]. The temporal interplay between muscle and adipose tissues requires integrated assessment approaches that evaluate their synergistic effects on healthy aging, with the muscle‐to‐fat ratio (MFR) serving as a potential biomarker for both age‐related and body composition‐related physiological changes [8].

Although previous studies have established normative values for select muscle metrics, mainly grip strength, in specific populations [9, 10], cross‐national comparisons incorporating multiple dimensions of body composition and related biomarkers remain limited [11]. This gap is particularly significant given well‐documented ethnic variations in body composition [12], with Asian populations typically exhibiting lower muscle mass but potentially different functional and metabolic characteristics compared with Western populations [13]. These differences necessitate population‐specific reference values for accurate clinical assessment and intervention planning [14]. Nevertheless, comparative studies examining ethnic and racial differences in age‐related lipolytic processes that drive subcutaneous‐to‐visceral adipose redistribution remain largely unexplored, representing a critical knowledge gap in population‐specific mechanisms of muscle–adipose temporal interactions during aging [15].

Our prior work established age‐ and sex‐specific normative values for muscle health metrics across Asian populations [10], demonstrating significant variations from Western standards. However, normative values for age‐related changes in muscle health metrics, adiposity measures and associated biomarkers across ethnically diverse populations remain poorly characterized, representing a critical knowledge gap for understanding population‐specific aging trajectories. In particular, although muscle–adipose tissue interactions during aging likely exhibit distinct patterns across genetically diverse populations, standardized reference values and comparative data across different ethnic groups have not been systematically established. Therefore, we aimed to develop age‐ and sex‐specific normative values for muscle health metrics, adiposity measures and metabolic biomarkers across three ethnically diverse, nationally representative cohorts from the United Kingdom, the United States and Taiwan. By describing age‐ and sex‐specific patterns of muscle and adipose tissue parameters within each population and examining how these observed patterns varied across cohorts, we sought to establish population‐specific reference standards and characterize differences in age‐related body composition patterns that could inform clinical assessment and future mechanistic research into the biological basis of population differences in healthy aging.

## 2 Methods

### 2.1 Data Sources

This study analysed data from three cohorts in the United Kingdom, the United States and Taiwan, with a primary focus on individuals aged ≥ 45 years because midlife is a key period during which age‐related differences in body composition become more apparent [10]. The cohorts included the UK Biobank (UKB), the US National Health and Nutrition Examination Survey (NHANES) and the Nutrition and Health Survey in Taiwan (NAHSIT).

The UKB is a large‐scale biomedical database and research resource containing de‐identified genetic, lifestyle and health information, and biological samples from half a million participants. Between 2006 and 2010, UKB recruited healthy volunteers aged between 37 and 73 years living across England, Wales and Scotland for baseline assessment. In 2014, the UKB imaging study re‐invited 100 000 participants to undergo dual‐energy X‐ray absorptiometry (DXA) scans for measurement of muscle and fat distribution. The ethical approval of UKB was granted by the North West Multi‐centre Research Ethics Committee.

The US NHANES is a series of studies that provide nationally representative data to monitor the nutrition and health of the US population. Participants were drawn from the noninstitutionalized civilian population using a complex, stratified, multistage probability‐cluster sampling design, which involved both in‐home interviews and visits to mobile examination centres. Detailed information on the survey design, protocols and data collection methods is publicly available online [16]. Due to variations in data collection across different NHANES waves, our study included all eligible participants with DXA examination data from 1999 to 2006 for measurements of appendicular lean mass (ALM), total fat mass and total fat percentage. Participants with handgrip strength data were included from the 2011–2014 survey waves. For muscle‐related biomarkers, we collected C‐reactive protein (CRP) data from 1999 to 2010 and data for other biomarkers from 1999 to 2018. All analyses used the recommended sample weights to address oversampling of specific subpopulations and account for survey nonresponse, ensuring the findings were representative of the US noninstitutionalized civilian population. The ethical approval of NHANES was granted by the US National Center for Health Statistics Ethics Review Board [17]. All participants provided written informed consent.

Data from the NAHSIT were utilized to evaluate key measurements within the Taiwanese population. NAHSIT is a comprehensive, nationwide survey aimed at monitoring the dietary, nutritional and health status of Taiwanese citizens. The survey employed a multistage, stratified and clustered probability sampling design to ensure representation of Taiwan's civilian, noninstitutionalized population across all age groups. Data collection was conducted through face‐to‐face interviews in respondents' homes, with health measurements carried out at designated local sites or in specially equipped mobile units travelling across Taiwan. Details regarding the survey design, protocols and data collection methods are accessible online to the public. The most recent survey waves used in this study were conducted between 2013–2016 and 2017–2020. All participant data were anonymized to protect confidentiality and identity. The institutional review board of National Taiwan University approved the NAHSIT protocol (202307077RIND).

### 2.2 Measurements

The muscle health metrics assessed in this study comprised a set of muscle measurements (muscle mass and muscle strength) (Table 1), including ALM (kg), height‐adjusted ALM (kg/m^2^), body mass index (BMI)‐adjusted ALM (kg/kg/m^2^) and handgrip strength (kg). ALM, representing muscle mass in the arms and legs, was consistently measured via DXA across all three cohorts. ALM was calculated as the sum of lean mass in the upper and lower limbs. To account for variations in body size, height‐adjusted ALM was derived by dividing ALM by squared height, whereas BMI‐adjusted ALM was computed by normalizing ALM to BMI. Handgrip strength was assessed with participants seated, their elbows flexed at 90° and their forearms in a neutral position. Participants exerted maximal force on a calibrated dynamometer using their dominant hand while maintaining proper alignment between the hand and forearm. A minimum of two measurements was recorded, with the highest value used for analysis, except for UKB, where handgrip strength was measured once for each hand.

**TABLE 1:** Measurement methods for muscle, adiposity and biomarker components across UK Biobank, NHANES and NAHSIT cohorts.

Component	UK Biobank	US NHANES	Taiwan NAHSIT
Muscle health	*N* = 16 311 (women); 19 125 (men)	*N* = 4641 (women); 4516 (men) Handgrip: *N* = 2749 (women); 2578 (men)	*N* = 2456 (women); 2503 (men)
ALM	Measured using whole‐body DXA; ALM calculated as the sum of lean mass in both arms and legs	Measured using DXA; ALM derived as the sum of limb lean mass	Measured using DXA; ALM computed from limb lean mass
ALM/height^2^	ALM divided by height squared (height measured during physical exam)	ALM divided by height squared	ALM divided by height squared
ALM/BMI	ALM normalized to BMI	ALM normalized to BMI	ALM normalized to BMI
aMFR	ALM divided by total fat mass	ALM divided by total fat mass	ALM divided by total fat mass
tMFR	Total lean mass divided by total fat mass	Total lean mass divided by total fat mass	Total lean mass divided by total fat mass
Handgrip strength	Assessed once for each hand using a calibrated dynamometer; the maximal value taken	Assessed using a hand dynamometer; the highest of repeated trials recorded	Assessed in seated position with standardized elbow angle; the highest of ≥ 2 trials used
Adiposity	*N* = 16 311 (women); 19 125 (men)	*N* = 4641 (women); 4516 (men)	*N* = 2456 (women); 2503 (men)
Fat mass	Total fat mass measured using whole‐body DXA	Total fat mass measured using DXA	Total fat mass measured using DXA
Body fat percentage	DXA‐derived fat mass divided by total body weight	DXA‐derived fat mass divided by total body weight	DXA‐derived fat mass divided by total body weight
Biomarker	*N* = 609–8965 (women); 516–8944 (men)	*N* = 6736–14 473 (women); 6466–14 050 (men)	*N* = 2779–2800 (women); 2801–2813 (men)
CRP	Blood sample analysis in UKB biomarker panel	Measured from blood samples (1999–2010)	Measured from blood samples in NAHSIT labs
HbA1c	Measured in blood samples during clinical assessment	Measured from fasting blood samples (1999–2018)	Measured from blood samples in NAHSIT surveys
Glucose	Measured from venous blood sample	Measured from fasting blood	Measured from fasting blood samples
Lipid profile (TC, HDL, LDL, triglycerides)	Assayed from blood samples using UKB standardized laboratory methods	Measured in fasting blood as part of NHANES clinical chemistry	Measured in blood samples collected in NAHSIT
Ketone bodies	Quantified from serum; available only in UKB metabolomic panel β‐hydroxybutyrate *N* = 8850 (women); 8926 (men) Triglycerides *N* = 8850 (women); 8926 (men)	—	—
IGF‐1	Measured from serum using UKB biochemical assays	—	—
Inflammatory cytokines	Measured using Olink Explore 3072 proteomic platform (NPX units)	—	—

Abbreviations: ALM, appendicular lean mass; ALM/BMI, BMI‐adjusted appendicular lean mass; ALM/height^2^, height‐adjusted appendicular lean mass; aMFR, appendicular muscle‐to‐fat ratio; BMI, body mass index; CRP, C‐reactive protein; DXA, dual‐energy X‐ray absorptiometry; HbA1c, haemoglobin A1c; HDL, high‐density lipoprotein; IGF‐1, insulin‐like growth factor 1; LDL, low‐density lipoprotein; NAHSIT, Nutrition and Health Survey in Taiwan; NHANES, US National Health and Nutrition Examination Survey; TC, total cholesterol; tMFR, total muscle‐to‐fat ratio; UKB, UK Biobank.

To further characterize body composition, total fat mass (kg) and body fat percentage (%) (Table 1), measured using DXA, were also reported. Body fat percentage was calculated as the ratio of total fat mass to total body weight and expressed as a percentage. MFRs (including appendicular MFR [aMFR] and total MFR [tMFR]) were also computed to provide insights into body composition. The aMFR was calculated by dividing ALM by total body fat mass, whereas the tMFR was obtained by dividing total body lean mass by total body fat mass.

In addition to assessments of muscle and fat mass, the study evaluated several biomarkers to examine the metabolic, cardiovascular and inflammatory implications of muscle health (Table 1). These biomarkers included CRP (mg/dL), glycated haemoglobin (HbA1c, %), fasting blood glucose (mg/dL), total cholesterol (mg/dL), high‐density lipoprotein cholesterol (HDL‐C, mg/dL), low‐density lipoprotein cholesterol (LDL‐C, mg/dL) and triglycerides (TG, mg/dL). Blood samples were collected as part of health examinations in each cohort. These biomarkers were selected based on their established associations with metabolic function, as well as their consistent measurement across the three cohorts. To investigate changes in MFR in the aging process, certain lipolysis‐related biomarkers existing uniquely in the UKB database were analysed. These included β‐hydroxybutyrate (BHB, mmol/L), acetoacetate (mmol/L), acetone (mmol/L) and insulin‐like growth factor‐1 (IGF‐1, nmol/L). Proteomic profiling using the antibody‐based Olink Explore 3072 PEA platform was performed on blood plasma samples from 54 219 UKB participants to analyse age‐related trends in lipolysis‐related biomarkers. Inflammatory cytokines, such as interleukin‐6 (IL‐6), interleukin‐1β (IL‐1β) and tumour necrosis factor (TNF), measured using the Olink platform were reported as normalized protein expression (NPX), a relative log2‐scaled measure of protein abundance. NPX values are expressed on a relative scale rather than as absolute concentrations; therefore, negative NPX values indicate lower relative abundance on the assay scale and should not be interpreted as negative protein concentrations.

### 2.3 Statistical Analyses

The study conducted descriptive analyses, including age‐ and sex‐specific normative values, cutoffs, and mean and 20th percentile values for metrics related to muscle health, adiposity and related biomarkers. Participants were stratified into 5‐year age groups (e.g., 45–49, 50–54 and 55–59), allowing for comparisons across different age subgroups.

To evaluate trends in these measurements, Joinpoint Trend Analysis Software (National Cancer Institute, Bethesda, MD, USA) was employed [18]. This analysis calculated age‐group percentage changes (APCs) to estimate the percent change per age group. The same regression models were utilized to assess differences in trends between sex subgroups. Joinpoint identifies time points where trends change significantly. Using age (in 5‐year intervals) as the timescale, Joinpoint regression begins with a straight line (0 joinpoints) and tests whether adding more joinpoints is statistically significant. The Monte Carlo permutation method was applied for significance testing, and the Bayesian information criterion (BIC) was used for model selection [18]. A *p*‐value of 0.05 or less was considered statistically significant. All analyses were performed using SAS, Version 9.4 (SAS Institute Inc., Cary, NC, USA).

### 2.4 Role of the Funding Source

The study sponsors were not involved in designing the study; collecting, analysing or interpreting the data; writing the report; or the decision to submit the paper for publication.

## 3 Results

### 3.1 Characteristics of Participants Across Three National Cohorts

The analysis included adults aged ≥ 45 years from the UKB, NHANES and NAHSIT. Sample sizes differed substantially across cohorts. The UKB included 35 436 participants (16 311 women; 19 125 men), NHANES included 9157 participants (4641 women; 4516 men), and NAHSIT included 4959 participants (2456 women; 2503 men) in the ALM analyses.

### 3.2 Age‐Specific Normative Values and Percentile Distributions

Tables S1–S8 present the detailed age‐ and sex‐specific normative values (5th, 25th, 50th, 75th and 95th percentiles) for muscle health metrics, adiposity measures and muscle‐related biomarkers across all three cohorts. Percentile distributions demonstrated consistent cross‐sectional patterns, with larger age‐group differences in the lower percentiles, indicating greater vulnerability in subpopulations with lower baseline values (Figures S3–S8). Adiposity percentile distributions revealed population‐specific patterns, with Western cohorts (UKB, NHANES) showing greater variability in fat mass across age groups compared with the Taiwanese cohort; the highest percentiles (75th–95th) in Western populations showed pronounced midlife peaks and lower values in later age groups, whereas lower percentiles remained relatively stable until advanced age (Figures S5 and S6). Biochemical marker percentiles showed coordinated age‐related differences across all populations, with the most pronounced values in upper percentiles at older ages, and earlier concentration of unfavourable glycemic values in higher percentiles particularly in the Taiwanese cohort (Figures S7 and S8).

### 3.3 Sex‐ and Age‐Specific Muscle Health Metrics

#### 3.3.1 ALM

**FIGURE 1:**
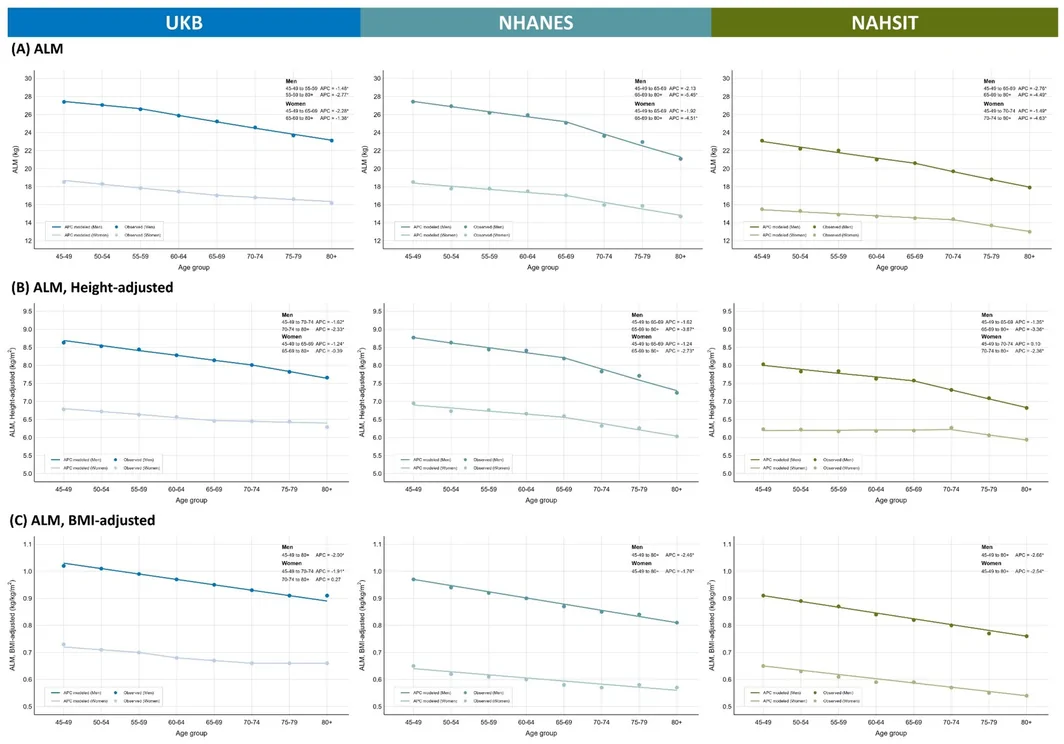
Age‐ and sex‐specific normative values for ALM and related metrics.

ALM was lower in older age groups across all cohorts in both sexes, with larger cross‐sectional differences across age groups in men than in women (Figure 1A; Tables S1 and S2). In the UKB, mean ALM was lower in older age groups, from 27.4 to 23.1 kg in men (−15.7%) and from 18.5 to 16.2 kg in women (−12.4%), with a steeper cross‐sectional pattern observed from ages 55–59 onward. Among UKB men, the 25th percentile of ALM was 24.3 kg at ages 45–49 and 21.2 kg at ages 80+, whereas the median was 26.7 kg in the youngest and 23.0 kg in the oldest age group; the corresponding values in women were 16.4 versus 14.5 kg at the 25th percentile and 18.1 versus 15.9 kg at the median (Tables S1 and S2). In NHANES, larger cross‐sectional differences across age groups were observed (men: −23.0%; women: −20.5%), with non‐linear patterns in later age groups, particularly after age 65. In NHANES women, mean ALM was 18.5 kg at ages 45–49 and 14.7 kg at ages 80+, with the largest between‐group difference observed between adjacent age groups 65–69 and 70–74 (mean: 17.0 vs. 16.0 kg) and between 70–74 and 80+ (16.0 vs. 14.7 kg). In NHANES men, mean ALM was 27.4 kg at ages 45–49 and 21.1 kg at ages 80+ (Tables S1 and S2). NAHSIT showed comparable cross‐sectional differences across age groups (men: −22.5%; women: −15.5%), with mean ALM in NAHSIT women of 15.5 kg at ages 45–49 and 13.1 kg at ages 80+, and in NAHSIT men 23.1 versus 17.9 kg.

#### 3.3.2 Height‐Adjusted and BMI‐Adjusted ALM

Height‐adjusted ALM (ALM/height^2^ [2]) showed smaller cross‐sectional differences across age groups than absolute ALM across all cohorts (Figure 1B, Tables S1 and S2). In the UKB, the differences between younger and older age groups were 11.2% in men and 7.2% in women, with mean height‐adjusted ALM of 8.63 kg/m^2^ in UKB men at ages 45–49 and 7.66 kg/m^2^ at ages 80+, and 6.78 versus 6.29 kg/m^2^ in UKB women. Relatively similar values across older age groups in UKB women (mean height‐adjusted ALM of 6.44 kg/m^2^ at ages 75–79 vs. 6.29 kg/m^2^ at ages 80+), suggesting potential masking effects of height loss. NHANES and NAHSIT showed larger cross‐sectional differences across age groups, particularly in men, with steeper patterns after age 70. In NHANES men, mean height‐adjusted ALM was 8.77 kg/m^2^ at ages 45–49 and 7.24 kg/m^2^ at ages 80+ (−17.5%); the corresponding values in NAHSIT men were 8.03 and 6.82 kg/m^2^ (−15.1%) (Tables S1 and S2).

BMI‐adjusted ALM is presented in Figure 1C and Tables S1 and S2. Differences between younger and older age groups ranged from 10.8% to 16.5% in men and from 9.6% to 16.9% in women across cohorts, with the largest age‐group differences observed from midlife onward. In UKB men, mean BMI‐adjusted ALM was 1.02 kg/kg/m^2^ at ages 45–49 and 0.91 kg/kg/m^2^ at ages 80+ (−10.8%), whereas in NHANES men the cross‐sectional difference was larger, from 0.97 to 0.81 kg/kg/m^2^ (−16.5%). Among women, BMI‐adjusted ALM was 0.73 versus 0.66 kg/kg/m^2^ in the UKB (−9.6%) and 0.65 versus 0.57 kg/kg/m^2^ in NHANES (−12.3%).

#### 3.3.3 MFRs

**FIGURE 2:**
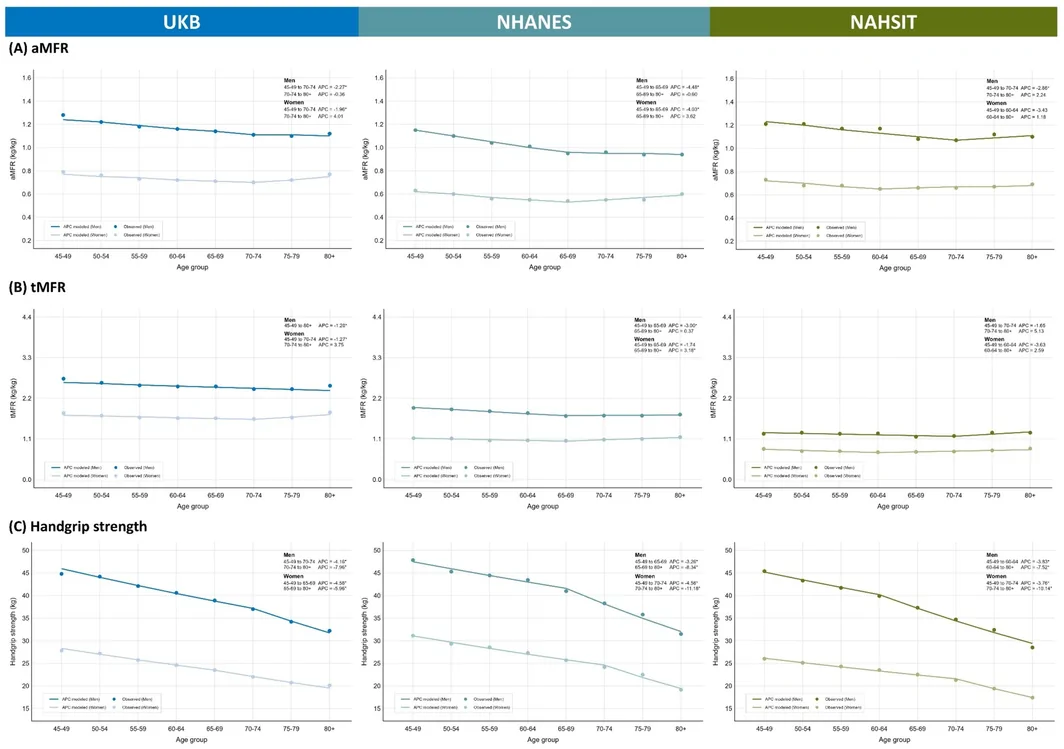
Age‐ and sex‐specific normative values for MFR and handgrip strength.

Both appendicular and total MFRs (aMFR, tMFR) exhibited non‐linear age‐related patterns that are distinct from muscle or fat measures alone (Figure 2A,B, Tables S1 and S2). In UKB and NHANES, MFRs were lower across mid‐to‐late adulthood, with relatively higher values in the oldest age groups, particularly among women. In UKB women, mean aMFR was 0.79 kg/kg at ages 45–49, reached its lowest value of 0.70 kg/kg at ages 70–74 and was 0.77 kg/kg at ages 80+. A similar non‐linear pattern was observed for tMFR in UKB women, which was 1.80 kg/kg at ages 45–49, 1.64 kg/kg at ages 70–74 and 1.80 kg/kg at ages 80+. In NHANES women, the aMFR at ages 80+ (0.60 kg/kg) was similar to that of the youngest age group (0.63 kg/kg at ages 45–49), reflecting this same non‐linear pattern (Tables S1 and S2). NAHSIT showed the most stable MFR patterns across age groups, with relatively small differences across age groups and slightly higher values in late life. In NAHSIT women, mean aMFR ranged from 0.73 kg/kg at ages 45–49 to 0.65 kg/kg at ages 60–64, with values of 0.69 kg/kg at ages 80+, and tMFR showed similarly modest variation (0.83 at ages 45–49, 0.74 at ages 60–64 and 0.84 at ages 80+) (Tables S1 and S2), suggesting population‐specific differences in body composition aging.

#### 3.3.4 Handgrip Strength

Handgrip strength was lower in older age groups across all cohorts and in both sexes (Figure 2C, Tables S1 and S2). Differences between younger and older age groups ranged from 28.6% to 38.7%, with larger age‐group differences typically observed after age 65. In UKB women, mean handgrip strength was 28 kg at ages 45–49 and 20 kg at ages 80+ (−28.6%); the corresponding values in UKB men were 45 and 32 kg (−28.9%). NHANES showed larger cross‐sectional differences, with mean handgrip strength of 31 versus 19 kg in women (−38.7%) and 48 versus 31 kg in men (−35.4%). In NAHSIT, mean handgrip strength was 26 kg at ages 45–49 and 17 kg at ages 80+ in women (−34.6%) and 45 versus 28 kg in men (−37.8%) (Tables S1 and S2).

#### 3.3.5 Adiposity Measures: Fat Mass and Fat Percentage

**FIGURE 3:**
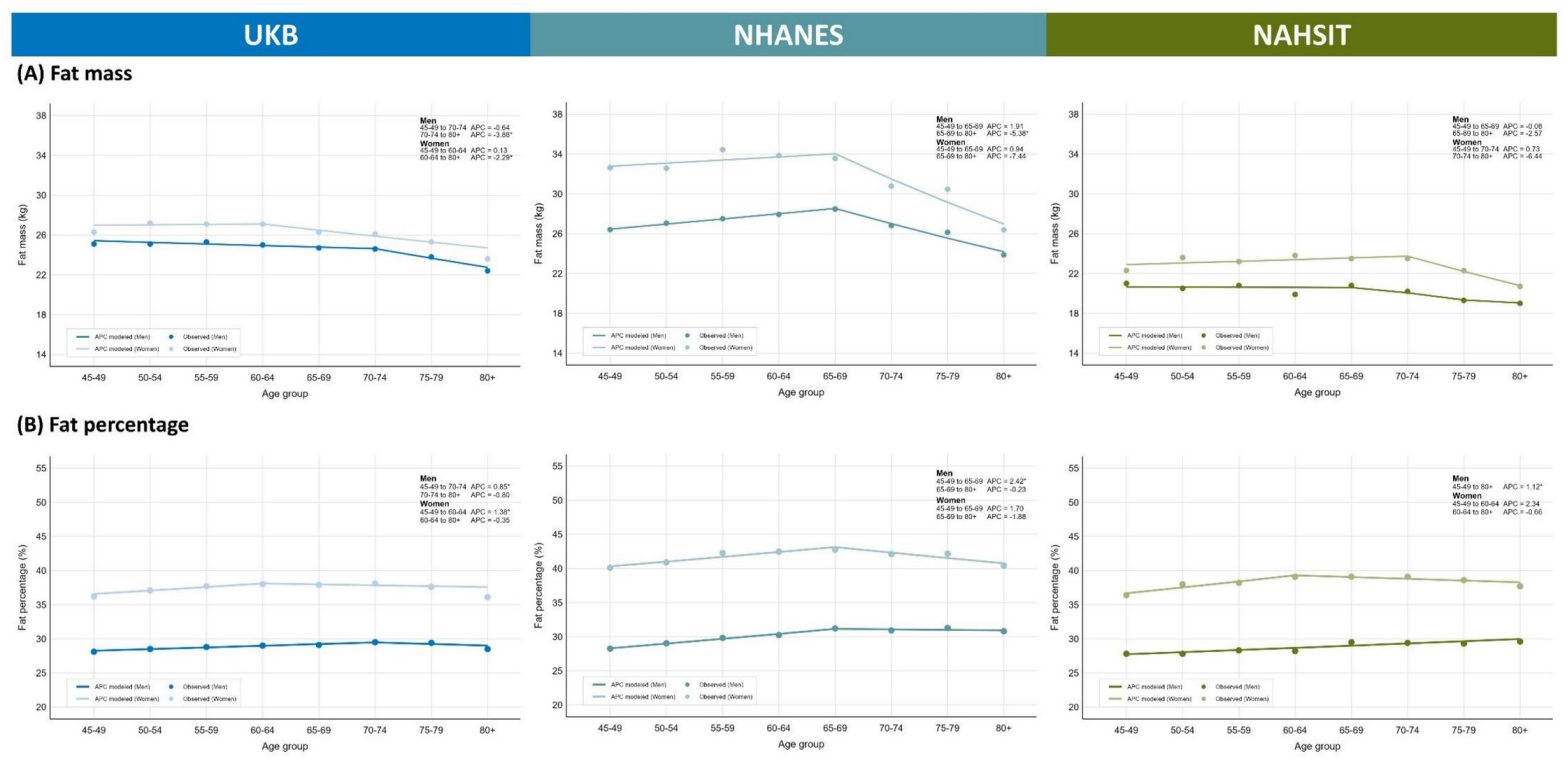
Age‐ and sex‐specific normative values for adiposity measures.

Fat mass and fat percentage followed cohort‐specific trajectories that are distinct from muscle measures (Figure 3, Tables S3 and S4). In UKB and NHANES, fat mass was higher in midlife age groups and lower in older age groups, whereas fat percentage was higher across older age groups before reaching a plateau or showing slightly lower values in the oldest groups. NAHSIT showed smaller cross‐sectional differences across age groups in midlife and late life, with fat percentage being higher in older age groups.

### 3.4 Inflammatory and Metabolic Biomarkers

**FIGURE 4:**
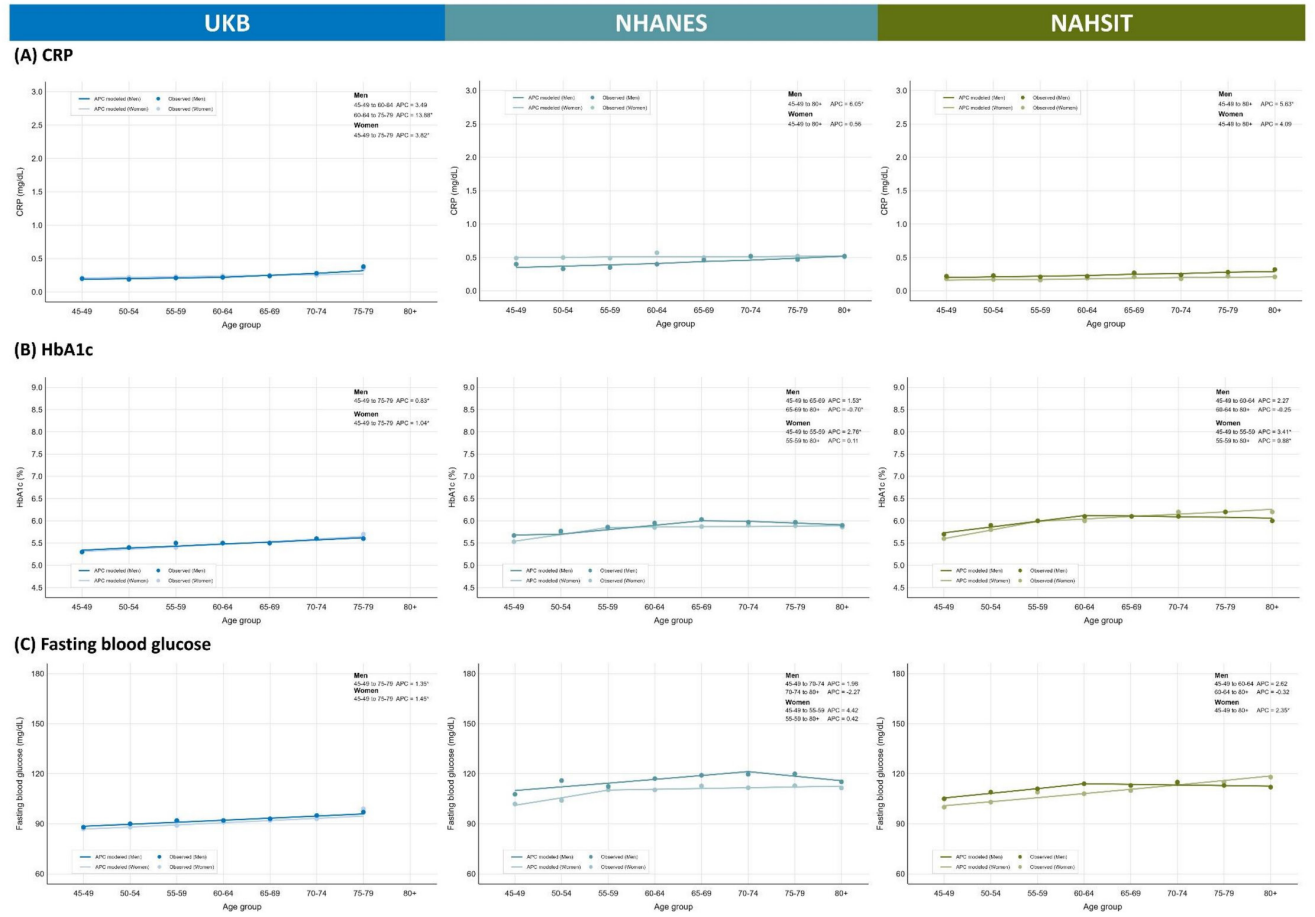
Age‐ and sex‐specific normative values for inflammatory and metabolic biomarkers.

Inflammatory and metabolic markers showed age‐related differences across cohorts (Figure 4, Tables S5 and S6). CRP was higher in older age groups in all populations, with larger cross‐sectional differences across age groups in NHANES and NAHSIT. In UKB women, median CRP was 0.09 mg/dL at ages 45–49 and 0.15 mg/dL at ages 75–79, whereas in UKB men the median was 0.10 versus 0.09 mg/dL over the same range. In NHANES, median CRP in women was 0.25 mg/dL at ages 45–49 and 0.23 mg/dL at ages 80+, and in men 0.16 versus 0.23 mg/dL. NAHSIT showed the largest relative cross‐sectional difference, with median CRP in women being 0.10 mg/dL at ages 45–49 and 0.12 mg/dL at ages 80+, and the 75th percentile being 0.16 versus 0.29 mg/dL (Tables S5 and S6). Glycemic markers (HbA1c and fasting glucose) were higher in older age groups. Median HbA1c in UKB women was 5.2% at ages 45–49 and 5.6% at ages 70–74; in NHANES women, 5.3% versus 5.7% at ages 80+. Median fasting glucose was consistently higher at all ages in NHANES women and NAHSIT women: (NHANES) 97 mg/dL at ages 45–49 and 103 mg/dL at ages 80+, and (NAHSIT) 95 mg/dL at ages 45–49 and 103 mg/dL at ages 80+ (Tables S5 and S6). Lipid profiles showed sex‐ and cohort‐specific midlife peaks and lower values in later age groups (Figures S1 and S2).

The UKB data on specialized metabolic and inflammatory markers (Tables S7 and S8, Figure S2) revealed that ketone bodies were modestly higher in older age groups in both sexes, whereas IGF‐1 was substantially lower in older age groups. Inflammatory cytokines were generally higher in older age groups, with IL‐6 showing the most pronounced age‐group differences, particularly in men. In UKB men, median IL‐6 (NPX) was −0.10 at ages 45–49 and +0.37 NPX at ages 65–69 and +0.38 at ages 70–74; in women, the corresponding values were −0.11, +0.15 and +0.26 NPX, respectively (Tables S7 and S8).

## 4 Discussion

This cross‐national analysis highlights substantial population‐specific differences in age‐related body composition patterns and supports the value of population‐specific approaches to healthy aging research and clinical practice. The observed ethnic variations in muscle‐related measures, adiposity patterns and inflammatory biomarkers may have important implications for clinical assessment frameworks, which currently rely largely on Western‐derived reference standards and may not fully capture variation in non‐Western populations. The preservation of favourable MFRs despite lower absolute muscle mass in Asian populations, coupled with distinct patterns of fat accumulation and metabolic marker profiles, suggests that current clinical assessment approaches may overemphasize tissue quantity while undervaluing metabolic efficiency and functional integration. These findings reveal fundamental gaps in our understanding of how genetic, environmental and lifestyle factors interact to shape body composition changes and inflammatory aging processes across diverse populations. The differences observed between BMI‐adjusted and height‐adjusted muscle mass measurements, together with population‐specific biomarker patterns, suggest that conventional assessment approaches may not fully capture clinically relevant age‐related differences. As global populations age rapidly with increasing ethnic diversity, these discoveries establish an urgent imperative for developing precision medicine strategies that account for population‐specific biological and metabolic characteristics rather than applying one‐size‐fits‐all interventions.

The systematic differences observed across the three cohorts may reflect contributions from genetic, environmental and lifestyle factors that are associated with age‐related body composition patterns. The steeper cross‐sectional age‐group differences observed earlier in Taiwanese men may reflect several interconnected factors. Genetic polymorphisms affecting muscle protein synthesis and degradation pathways varied across populations, potentially influencing the timing and rate of age‐related muscle atrophy. Additionally, occupational patterns and physical activity levels throughout the lifespan differed substantially between Asian and Western populations, with implications for muscle mass maintenance. The distinct adiposity patterns observed across cohorts—with Western populations showing earlier fat mass peaks (ages 55–59) compared with Taiwanese participants (ages 60–64)—reflected differences in dietary patterns, metabolic programming and lifestyle transitions [19]. The Western cohorts' earlier peak fat accumulation could be attributed to the ‘midlife obesity crisis’ characterized by sedentary work environments, caloric excess and metabolic dysregulation associated with urbanized lifestyles [20]. Importantly, the preservation of favourable MFRs in the Taiwanese cohort despite lower absolute muscle mass suggested that body composition efficiency, rather than quantity, may be the critical determinant of metabolic health. This finding aligns with population differences in insulin sensitivity and cardiovascular risk profiles, where Asian populations demonstrate metabolic dysfunction at lower BMI thresholds, potentially due to different muscle quality or metabolic characteristics rather than simply muscle quantity [21].

The observation that BMI‐adjusted ALM showed larger differences across age groups than height‐adjusted values provides an important methodological perspective. BMI adjustment may better capture age‐related differences in muscle measures by accounting for overall body size and may be useful for identifying individuals with lower relative muscle mass [22]. Furthermore, the unexpected late‐life increases in MFRs, particularly pronounced in women aged 80+, suggested potential survival selection effects that have not been systematically documented in previous normative studies. Perhaps most intriguingly, despite having substantially lower absolute muscle mass values, Taiwanese participants maintained similar or even more favourable MFRs compared with Western populations. This finding provided insights into the ‘obesity paradox’ of Asian populations and suggested that relative body composition, rather than absolute tissue quantities, was more physiologically relevant for metabolic health outcomes [23]. The relationship between inflammatory markers and metabolic dysfunction showed population‐specific patterns that may reflect fundamental ethnic and dietary differences between Asian and Western populations.

Our findings significantly extend existing literature on age‐related body composition changes while highlighting important gaps in current understanding. Previous studies, primarily focused on Western populations, reported relatively linear muscle mass declines of approximately 1%–2% per year after age 50 [24]. Our joinpoint analysis suggested that age‐related patterns were more complex, with non‐linear differences across age groups that varied across populations. The higher inflammatory marker levels observed in older age groups across all cohorts are consistent with the ‘inflammaging’ hypothesis [25] and suggest that chronic low‐grade inflammation may be relevant to age‐related differences in muscle‐related measures. However, our cross‐population comparison revealed important nuances—the NAHSIT cohort demonstrated more pronounced late‐life inflammatory responses despite generally lower baseline levels, suggesting population‐specific inflammatory aging patterns that may influence the timing and severity of muscle loss.

Our analysis suggests complex relationships between muscle and adipose tissues that extend beyond simple inverse relationships. The non‐linear adiposity patterns—characterized by midlife increases followed by late‐life decreases—suggest complex metabolic reprogramming during aging. The earlier fat mass peak observed in Western populations (ages 55–65) followed by subsequent declines may reflect the transition from anabolic to catabolic aging phases, where metabolic efficiency diminishes and total energy expenditure decreases [26]. The modest but consistent increases in ketone bodies (β‐HB, acetoacetate and acetone) with advancing age provide molecular evidence of enhanced lipolytic activity in older adults. This metabolic shift toward fat oxidation may represent an adaptive response to declining glucose utilization efficiency, supporting that aging involves fundamental changes in substrate utilization preferences [27]. The observation that these changes occurred earlier and more prominently in Western cohorts aligned with their earlier onset of fat mass decline. Particularly intriguing were the relatively higher MFR values in late life, especially pronounced in women aged 80+. This pattern suggested that the oldest survivors may represent a metabolically distinct subgroup with preserved muscle‐fat balance—potentially indicating that favourable body composition contributes to exceptional longevity in centenarian studies [28]. This ‘survival selection’ effect may partly explain why population‐based studies often underestimate the severity of age‐related body composition changes in the general population. The tissue redistribution patterns also revealed important sex differences. Women showed more stable MFRs in advanced age, possibly reflecting oestrogen‐mediated protective effects on muscle mass or differences in fat distribution patterns [29]. The relatively stable aMFR values in older women, despite lower absolute muscle mass in older age groups, suggested that selective fat loss from extremities may partially compensate for muscle atrophy.

The age‐related increases in HbA1c and glucose levels across all cohorts, coupled with declining muscle mass, supported the concept of ‘muscle as metabolic sink’ where reduced muscle quantity impaired glucose disposal capacity [30]. The earlier and more pronounced elevations in glycemic markers in the Taiwanese cohort, despite lower absolute adiposity, may reflect the previously described ‘thin‐fat’ phenotype common in Asian populations [31]. Particularly intriguing is the late‐life reversal in MFRs decline, especially pronounced in women aged 80+. This pattern suggests that the oldest survivors may represent a metabolically distinct subgroup with preserved muscle‐fat balance—potentially indicating that favourable body composition contributes to exceptional longevity in centenarian studies [32].

The inverse relationship between declining IGF‐1 levels and increasing inflammatory cytokines observed across all cohorts provides mechanistic insights into age‐related anabolic resistance [33]. The 20%–24% decline in IGF‐1 from ages 45–49 to 75–79 parallels the acceleration in muscle mass loss, supporting that declining growth factor signalling contributes to age‐related muscle atrophy [34]. This relationship appeared most pronounced in the Western cohorts, potentially explaining the later but more severe muscle loss patterns observed in these populations. Our biomarker findings also revealed important metabolic implications of body composition changes. The age‐related increases in HbA1c and glucose levels across all cohorts, coupled with declining muscle mass, supported the concept of ‘muscle as metabolic sink’ where reduced muscle quantity impaired glucose disposal capacity [35].

These metabolic differences may be partially explained by distinct dietary patterns characteristic of Asian populations. Asian diets were characteristically high in carbohydrates, often comprising 60% or more of total energy intake compared with Western populations [36], which may explain the higher baseline HbA1c observed in Taiwanese participants. Higher carbohydrate intake, particularly from refined grains with elevated glycemic index, is recognized as a major dietary risk factor contributing to metabolic dysfunction in South Asian populations. However, paradoxically, these participants showed less pronounced inflammatory marker elevations despite their altered glycemic profiles, suggesting metabolic adaptations that may provide protection against inflammatory aging. This apparent metabolic paradox may be reconciled by considering the broader dietary context of Asian populations. Recent evidence demonstrated that plant‐based dietary patterns, which are more prevalent in Asian populations, were associated with reduced all‐cause mortality and favourable health outcomes among older Chinese adults [37]. The favourable effects of plant‐rich diets may counterbalance the potential metabolic disadvantages of higher carbohydrate intake, representing a complex interplay between carbohydrate quality and source rather than quantity alone. Additionally, Chinese centenarians demonstrated unique glycemic variability patterns that may contribute to their exceptional longevity despite altered glucose metabolism [38]. This suggested that earlier metabolic adaptation to higher carbohydrate loads, combined with the protective benefits of plant‐based dietary components, may trigger different but potentially more sustainable aging mechanisms that attenuate inflammatory aging pathways.

### 4.1 Study Strengths and Limitations

This study provided several important strengths that enhance the validity and generalizability of our findings. The analysis of three large, nationally representative cohorts spanning diverse ethnic populations offered unprecedented scope for understanding population‐specific aging patterns, addressing recent calls for more inclusive sarcopenia research [39]. The use of joinpoint regression analysis to identify critical turning points in body composition trajectories represented a methodological advancement over previous linear modelling approaches, providing clinically relevant insights into when interventions might be most effective [40]. However, several limitations warranted consideration. First, the cross‐sectional design precluded direct assessment of within‐individual changes over time, potentially leading to misinterpretation of age‐related trends due to cohort effects and survival bias. However, the implementation of multiple longitudinal studies spanning decades across diverse geographic regions and ethnic populations is methodologically and logistically challenging; consequently, the present approach represents the most viable methodological alternative. Second, the relatively small sample sizes in the oldest age cohorts (80+) may have compromised the reliability of reference values for these populations, whereas selective survival bias inherent to this age group may have introduced systematic error into the conclusions. Additionally, the biomarker analyses were constrained by differential availability across cohorts, limiting our ability to fully characterize the biological mechanisms underlying population‐specific aging patterns.

## 5 Conclusions

This cross‐national analysis identified population‐specific age‐related patterns in body composition across the United Kingdom, the United States and Taiwanese cohorts. In particular, the preservation of favourable MFRs in Asian populations despite lower absolute muscle mass challenged current paradigms and suggested that relative body composition efficiency was more clinically relevant than absolute tissue quantities. The identification of non‐linear age‐related patterns in muscle measures indicates that the age ranges at which cross‐sectional differences become more apparent may vary across populations. These normative values established essential reference standards for early detection of sarcopenia and highlighted the urgent need for ethnically tailored approaches to healthy aging. As global populations aged rapidly, these findings provided a crucial foundation for developing precision medicine strategies that could be optimally timed and population‐specific to promote healthy longevity worldwide.

## Funding

This research was funded by the Taiwan National Science and Technology Council (NSTC 113‐2321‐B‐A49‐028 and NSTC 114‐2321‐B‐A49‐012), and the Interdisciplinary Research Center for Healthy Longevity of National Yang Ming Chiao Tung University from The Featured Areas Research Center Program within the framework of the Higher Education Sprout Project by the Ministry of Education (MOE) in Taiwan. The funding source had no role in conducting this study, including the study design, data collection and analysis, manuscript preparation and review, or the decision to submit the manuscript for publication.

## Conflicts of Interest

The authors declare no conflicts of interest.

## Supporting information


**Table S1:** Age‐specific normative values for metrics of muscle health among women.
**Table S2:** Age‐specific normative values for metrics of muscle health among men.
**Table S3:** Age‐specific normative values for adiposity measures among women.
**Table S4:** Age‐specific normative values for adiposity measures among men.
**Table S5:** Age‐specific normative values for muscle‐related biomarkers among women.
**Table S6:** Age‐specific normative values for muscle‐related biomarkers among men.
**Table S7:** Age‐specific normative values for lipid metabolism‐related biomarkers among women.
**Table S8:** Age‐specific normative values for lipid metabolism‐related biomarkers among men.
**Figure S1:** Age‐specific normative values for lipid profiles.
**Figure S2:** Age‐ and sex‐specific normative values for metrics of lipid metabolism‐related biomarkers in UKB.
**Figure S3:** Age‐specific percentiles for muscle health metrics among women.
**Figure S4:** Age‐specific percentiles for muscle health metrics among men.
**Figure S5:** Age‐ and sex‐specific normative values for adiposity measures among women.
**Figure S6:** Age‐ and sex‐specific normative values for adiposity measures among men.
**Figure S7:** Age‐specific percentiles for biochemistry measures among women.
**Figure S8:** Age‐specific percentiles for biochemistry measures among men.

## Data Availability

The data that support the findings of this study are available on request from the corresponding author. The data are not publicly available due to privacy or ethical restrictions.
