# Supplementary material for: Muscle–Adipose Aging Dynamics and Inflammaging Patterns Across Ethnically Diverse Populations: Exploring Potential Roles of Lipolysis
Source: J Cachexia Sarcopenia Muscle. 2026 Jun 4;17(3):e70316. doi: 10.1002/jcsm.70316 (PMC13581085; doi:10.1002/jcsm.70316)
Supplement: Supplementary file 1 [file JCSM-17-e70316-s001.docx]

**Supplementary materials**

**Supplementary Tables**

Supplementary Table 1. Age-specific normative values for metrics of muscle health among women

Supplementary Table 2. Age-specific normative values for metrics of muscle health among men

Supplementary Table 3. Age-specific normative values for adiposity measures among women

Supplementary Table 4. Age-specific normative values for adiposity measures among men

Supplementary Table 5. Age-specific normative values for muscle-related biomarkers among women

Supplementary Table 6. Age-specific normative values for muscle-related biomarkers among men

Supplementary Table 7. Age-specific normative values for lipid metabolism-related biomarkers among women

Supplementary Table 8. Age-specific normative values for lipid metabolism-related biomarkers among men

**Supplementary Figures**

Supplementary Figure 1. Age- specific normative values for lipid profiles

Supplementary Figure 2. Age- and sex-specific normative values for metrics of lipid metabolism-related biomarkers in UKB

Supplementary Figure 3. Age- specific percentiles for muscle health metrics among women

Supplementary Figure 4. Age- specific percentiles for muscle health metrics among men

Supplementary Figure 5. Age- and sex-specific normative values for adiposity measures among women

Supplementary Figure 6. Age- and sex-specific normative values for adiposity measures among men

Supplementary Figure 7. Age- specific percentiles for biochemistry measures among women

Supplementary Figure 8. Age- specific percentiles for biochemistry measures among men

**Supplementary Table 1. Age-specific normative values for metrics of muscle health among women**

A. **UK biobank**

|  | Observations | Percentiles | | | | | Mean | 20th |
| --- | --- | --- | --- | --- | --- | --- | --- | --- |
|  |  | **5th** | **25th** | **50th** | **75th** | **95th** |  |  |
| **ALM (kg)** | | | | | | | | |
| 45-49 | 432 | 14.4 | 16.4 | 18.1 | 20.3 | 24.2 | 18.5 | 16.0 |
| 50-54 | 2402 | 14.2 | 16.3 | 18.0 | 19.9 | 23.5 | 18.3 | 15.9 |
| 55-59 | 3519 | 14.0 | 15.9 | 17.5 | 19.4 | 22.7 | 17.8 | 15.6 |
| 60-64 | 4156 | 13.8 | 15.7 | 17.2 | 19.0 | 22.2 | 17.5 | 15.3 |
| 65-69 | 4467 | 13.4 | 15.4 | 16.8 | 18.4 | 21.4 | 17.0 | 15.0 |
| 70-74 | 3569 | 13.4 | 15.2 | 16.6 | 18.2 | 21.1 | 16.8 | 14.8 |
| 75-79 | 1555 | 13.2 | 15.1 | 16.4 | 18.0 | 20.6 | 16.6 | 14.7 |
| 80+ | 211 | 12.7 | 14.5 | 15.9 | 17.6 | 20.5 | 16.2 | 14.1 |
| **ALM, Height-adjusted (kg/m^2^)** | | | | | | | | |
| 45-49 | 432 | 5.54 | 6.11 | 6.64 | 7.33 | 8.52 | 6.78 | 6.02 |
| 50-54 | 2402 | 5.46 | 6.09 | 6.61 | 7.19 | 8.41 | 6.72 | 5.97 |
| 55-59 | 3519 | 5.36 | 6.02 | 6.52 | 7.11 | 8.31 | 6.63 | 5.90 |
| 60-64 | 4156 | 5.37 | 5.98 | 6.45 | 7.02 | 8.17 | 6.57 | 5.87 |
| 65-69 | 4467 | 5.29 | 5.91 | 6.36 | 6.91 | 7.99 | 6.46 | 5.80 |
| 70-74 | 3569 | 5.31 | 5.91 | 6.36 | 6.93 | 7.82 | 6.45 | 5.80 |
| 75-79 | 1555 | 5.34 | 5.92 | 6.38 | 6.87 | 7.87 | 6.44 | 5.80 |
| 80+ | 211 | 5.02 | 5.69 | 6.27 | 6.72 | 7.78 | 6.29 | 5.56 |
| **ALM, BMI-adjusted (kg/kg/m^2^)** | | | | | | | | |
| 45-49 | 432 | 0.57 | 0.66 | 0.72 | 0.78 | 0.90 | 0.73 | 0.65 |
| 50-54 | 2402 | 0.55 | 0.64 | 0.71 | 0.78 | 0.89 | 0.71 | 0.63 |
| 55-59 | 3519 | 0.55 | 0.63 | 0.69 | 0.76 | 0.86 | 0.70 | 0.61 |
| 60-64 | 4156 | 0.54 | 0.61 | 0.67 | 0.74 | 0.85 | 0.68 | 0.60 |
| 65-69 | 4467 | 0.53 | 0.61 | 0.67 | 0.73 | 0.83 | 0.67 | 0.59 |
| 70-74 | 3569 | 0.53 | 0.60 | 0.66 | 0.72 | 0.81 | 0.66 | 0.59 |
| 75-79 | 1555 | 0.53 | 0.60 | 0.65 | 0.71 | 0.81 | 0.66 | 0.58 |
| 80+ | 211 | 0.53 | 0.59 | 0.65 | 0.71 | 0.84 | 0.66 | 0.58 |
| **aMFR (kg/kg)** | | | | | | | | |
| 45-49 | 432 | 0.47 | 0.60 | 0.73 | 0.90 | 1.29 | 0.79 | 0.57 |
| 50-54 | 2402 | 0.44 | 0.57 | 0.70 | 0.88 | 1.27 | 0.76 | 0.54 |
| 55-59 | 3519 | 0.44 | 0.56 | 0.68 | 0.84 | 1.22 | 0.73 | 0.53 |
| 60-64 | 4156 | 0.44 | 0.55 | 0.66 | 0.82 | 1.20 | 0.73 | 0.53 |
| 65-69 | 4467 | 0.44 | 0.55 | 0.66 | 0.81 | 1.15 | 0.71 | 0.53 |
| 70-74 | 3569 | 0.45 | 0.55 | 0.65 | 0.79 | 1.14 | 0.70 | 0.53 |
| 75-79 | 1555 | 0.46 | 0.56 | 0.67 | 0.81 | 1.14 | 0.72 | 0.54 |
| 80+ | 211 | 0.46 | 0.58 | 0.70 | 0.87 | 1.36 | 0.77 | 0.55 |
| **tMFR (kg/kg)** | | | | | | | | |
| 45-49 | 432 | 1.04 | 1.33 | 1.65 | 2.07 | 2.97 | 1.80 | 1.26 |
| 50-54 | 2402 | 0.97 | 1.27 | 1.60 | 2.03 | 2.96 | 1.73 | 1.21 |
| 55-59 | 3519 | 0.97 | 1.25 | 1.56 | 1.95 | 2.85 | 1.68 | 1.19 |
| 60-64 | 4156 | 0.97 | 1.24 | 1.51 | 1.90 | 2.90 | 1.66 | 1.19 |
| 65-69 | 4467 | 0.99 | 1.26 | 1.53 | 1.91 | 2.73 | 1.66 | 1.21 |
| 70-74 | 3569 | 1.00 | 1.26 | 1.51 | 1.86 | 2.73 | 1.64 | 1.21 |
| 75-79 | 1555 | 1.04 | 1.29 | 1.54 | 1.89 | 2.79 | 1.68 | 1.24 |
| 80+ | 211 | 1.02 | 1.33 | 1.62 | 2.12 | 3.31 | 1.80 | 1.27 |
| **Handgrip strength (kg)** | | | | | | | | |
| 45-49 | 432 | 18 | 24 | 28 | 32 | 38 | 28 | 22 |
| 50-54 | 2402 | 18 | 23 | 28 | 31 | 36 | 27 | 22 |
| 55-59 | 3519 | 16 | 22 | 26 | 30 | 35 | 26 | 20 |
| 60-64 | 4156 | 15 | 20 | 24 | 28 | 34 | 25 | 20 |
| 65-69 | 4467 | 14 | 20 | 24 | 28 | 32 | 24 | 19 |
| 70-74 | 3569 | 12 | 18 | 22 | 26 | 30 | 22 | 18 |
| 75-79 | 1555 | 10 | 18 | 20 | 24 | 30 | 21 | 16 |
| 80+ | 211 | 10 | 16 | 20 | 24 | 28 | 20 | 16 |

B. **NHANES**

|  |  | Observations | Percentiles | | | | | Mean | | 20th |
| --- | --- | --- | --- | --- | --- | --- | --- | --- | --- | --- |
|  |  |  | **5th** | **25th** | **50th** | **75th** | **95th** |  |  |  |
|  | **ALM (kg)** | | | | | | | | | |
|  | 45-49 | 747 | 12.9 | 15.6 | 17.7 | 20.8 | 26.1 | 18.5 | | 15.1 |
|  | 50-54 | 706 | 12.7 | 15.1 | 17.3 | 19.7 | 24.7 | 17.8 | | 14.7 |
|  | 55-59 | 487 | 12.6 | 15.0 | 17.2 | 20.0 | 25.2 | 17.8 | | 14.6 |
|  | 60-64 | 780 | 12.7 | 14.8 | 16.9 | 19.6 | 24.6 | 17.5 | | 14.3 |
|  | 65-69 | 628 | 12.2 | 14.5 | 16.5 | 18.8 | 23.6 | 17.0 | | 14.1 |
|  | 70-74 | 414 | 11.7 | 13.9 | 15.7 | 17.5 | 21.7 | 16.0 | | 13.5 |
|  | 75-79 | 310 | 12.1 | 13.7 | 15.2 | 17.8 | 20.8 | 15.9 | | 13.4 |
|  | 80+ | 569 | 11.0 | 12.7 | 14.4 | 16.1 | 20.2 | 14.7 | | 12.4 |
|  | **ALM, Height-adjusted (kg/m^2^)** | | | | | | | | | |
|  | 45-49 | 747 | 5.09 | 5.82 | 6.65 | 7.80 | 9.74 | 6.95 | | 5.73 |
|  | 50-54 | 706 | 4.88 | 5.84 | 6.50 | 7.41 | 9.23 | 6.73 | | 5.69 |
|  | 55-59 | 487 | 4.98 | 5.69 | 6.52 | 7.50 | 9.47 | 6.76 | | 5.50 |
|  | 60-64 | 780 | 4.99 | 5.70 | 6.46 | 7.35 | 9.16 | 6.66 | | 5.59 |
|  | 65-69 | 628 | 4.92 | 5.69 | 6.43 | 7.21 | 8.85 | 6.59 | | 5.55 |
|  | 70-74 | 414 | 4.73 | 5.54 | 6.22 | 6.91 | 8.36 | 6.32 | | 5.37 |
|  | 75-79 | 310 | 4.93 | 5.52 | 5.99 | 6.90 | 8.15 | 6.26 | | 5.31 |
|  | 80+ | 569 | 4.68 | 5.28 | 5.94 | 6.50 | 7.87 | 6.03 | | 5.28 |
|  | **ALM, BMI-adjusted (kg/kg/m^2^)** | | | | | | | | | |
|  | 45-49 | 747 | 0.50 | 0.58 | 0.64 | 0.70 | 0.82 | 0.65 | | 0.57 |
|  | 50-54 | 706 | 0.49 | 0.56 | 0.61 | 0.68 | 0.78 | 0.62 | | 0.55 |
|  | 55-59 | 487 | 0.47 | 0.55 | 0.60 | 0.66 | 0.76 | 0.61 | | 0.53 |
|  | 60-64 | 780 | 0.48 | 0.54 | 0.59 | 0.65 | 0.75 | 0.60 | | 0.53 |
|  | 65-69 | 628 | 0.46 | 0.53 | 0.58 | 0.63 | 0.71 | 0.58 | | 0.52 |
|  | 70-74 | 414 | 0.45 | 0.52 | 0.56 | 0.62 | 0.72 | 0.57 | | 0.51 |
|  | 75-79 | 310 | 0.46 | 0.52 | 0.57 | 0.62 | 0.72 | 0.58 | | 0.51 |
|  | 80+ | 569 | 0.45 | 0.51 | 0.56 | 0.62 | 0.72 | 0.57 | | 0.50 |
|  | **aMFR (kg/kg)** | | | | | | | | | |
|  | 45-49 | 747 | 0.40 | 0.49 | 0.58 | 0.72 | 1.06 | 0.63 | | 0.47 |
|  | 50-54 | 706 | 0.40 | 0.48 | 0.55 | 0.67 | 0.91 | 0.60 | | 0.46 |
|  | 55-59 | 487 | 0.38 | 0.46 | 0.52 | 0.63 | 0.87 | 0.56 | | 0.44 |
|  | 60-64 | 780 | 0.39 | 0.45 | 0.52 | 0.61 | 0.81 | 0.55 | | 0.44 |
|  | 65-69 | 628 | 0.38 | 0.45 | 0.52 | 0.60 | 0.79 | 0.54 | | 0.44 |
|  | 70-74 | 414 | 0.38 | 0.46 | 0.52 | 0.62 | 0.82 | 0.55 | | 0.44 |
|  | 75-79 | 310 | 0.39 | 0.46 | 0.52 | 0.59 | 0.81 | 0.55 | | 0.44 |
|  | 80+ | 569 | 0.41 | 0.48 | 0.56 | 0.67 | 0.92 | 0.60 | | 0.46 |
| **tMFR (kg/kg)** | | | | | | | | | | |
| 45-49 | | 747 | 0.93 | 1.16 | 1.39 | 1.73 | 2.50 | 1.50 | 1.12 | |
| 50-54 | | 706 | 0.93 | 1.15 | 1.34 | 1.61 | 2.22 | 1.44 | 1.11 | |
| 55-59 | | 487 | 0.89 | 1.11 | 1.27 | 1.52 | 2.20 | 1.36 | 1.06 | |
| 60-64 | | 780 | 0.93 | 1.09 | 1.26 | 1.51 | 1.98 | 1.34 | 1.06 | |
| 65-69 | | 628 | 0.89 | 1.10 | 1.26 | 1.48 | 1.95 | 1.32 | 1.05 | |
| 70-74 | | 414 | 0.93 | 1.10 | 1.28 | 1.51 | 2.09 | 1.36 | 1.08 | |
| 75-79 | | 310 | 0.95 | 1.14 | 1.28 | 1.48 | 2.04 | 1.36 | 1.10 | |
| 80+ | | 569 | 1.00 | 1.20 | 1.38 | 1.64 | 2.24 | 1.47 | 1.15 | |
|  | **Handgrip strength (kg)** | | | | | | | | | |
|  | 45-49 | 412 | 22 | 28 | 31 | 35 | 41 | 31 | | 27 |
|  | 50-54 | 440 | 21 | 26 | 30 | 32 | 39 | 29 | | 25 |
|  | 55-59 | 374 | 19 | 25 | 28 | 32 | 37 | 29 | | 25 |
|  | 60-64 | 455 | 20 | 24 | 27 | 30 | 35 | 27 | | 23 |
|  | 65-69 | 326 | 18 | 23 | 26 | 29 | 33 | 26 | | 22 |
|  | 70-74 | 297 | 15 | 21 | 24 | 27 | 32 | 24 | | 20 |
|  | 75-79 | 171 | 13 | 20 | 22 | 26 | 31 | 22 | | 18 |
|  | 80+ | 274 | 12 | 16 | 19 | 23 | 27 | 19 | | 15 |

C. **NAHSIT**

|  | Observations | Percentiles | | | | | | Mean | 20th |
| --- | --- | --- | --- | --- | --- | --- | --- | --- | --- |
|  |  | **5th** | **25th** | **50th** | **75th** | **95th** | |  |  |
| **ALM (kg)** | | | | | | | | | |
| 45-49 | 251 | 12.3 | 13.7 | 15.3 | 17.0 | 19.6 | | 15.5 | 13.4 |
| 50-54 | 274 | 12.0 | 13.7 | 15.1 | 16.6 | 19.8 | | 15.3 | 13.4 |
| 55-59 | 314 | 12.1 | 13.5 | 14.7 | 16.2 | 18.2 | | 14.9 | 13.3 |
| 60-64 | 318 | 11.3 | 13.1 | 14.4 | 16.1 | 18.8 | | 14.7 | 12.8 |
| 65-69 | 564 | 11.3 | 13.2 | 14.3 | 15.8 | 18.1 | | 14.5 | 12.8 |
| 70-74 | 360 | 11.5 | 12.9 | 14.3 | 15.6 | 17.4 | | 14.4 | 12.7 |
| 75-79 | 230 | 10.3 | 12.3 | 13.4 | 14.9 | 17.1 | | 13.7 | 12.0 |
| 80+ | 145 | 9.8 | 11.6 | 13.1 | 14.4 | 16.3 | | 13.1 | 11.1 |
| **ALM, Height-adjusted (kg/m^2^)** | | | | | | | | | |
| 45-49 | 251 | 4.96 | 5.57 | 6.13 | 6.76 | 8.03 | | 6.23 | 5.49 |
| 50-54 | 274 | 5.01 | 5.54 | 6.17 | 6.70 | 7.80 | | 6.22 | 5.46 |
| 55-59 | 314 | 4.96 | 5.66 | 6.14 | 6.67 | 7.54 | | 6.17 | 5.53 |
| 60-64 | 318 | 4.86 | 5.62 | 6.11 | 6.69 | 7.71 | | 6.18 | 5.52 |
| 65-69 | 564 | 4.92 | 5.68 | 6.14 | 6.71 | 7.50 | | 6.19 | 5.55 |
| 70-74 | 360 | 5.02 | 5.73 | 6.24 | 6.76 | 7.53 | | 6.27 | 5.63 |
| 75-79 | 230 | 4.89 | 5.50 | 5.99 | 6.56 | 7.55 | | 6.06 | 5.38 |
| 80+ | 145 | 4.65 | 5.56 | 5.94 | 6.34 | 7.14 | | 5.94 | 5.35 |
| **ALM, BMI-adjusted (kg/kg/m^2^)** | | | | | | | | | |
| 45-49 | 251 | 0.53 | 0.60 | 0.65 | 0.70 | 0.77 | | 0.65 | 0.59 |
| 50-54 | 274 | 0.51 | 0.57 | 0.63 | 0.67 | 0.76 | | 0.63 | 0.56 |
| 55-59 | 314 | 0.50 | 0.56 | 0.60 | 0.66 | 0.73 | | 0.61 | 0.55 |
| 60-64 | 318 | 0.48 | 0.55 | 0.59 | 0.64 | 0.70 | | 0.59 | 0.54 |
| 65-69 | 564 | 0.46 | 0.54 | 0.59 | 0.63 | 0.70 | | 0.59 | 0.53 |
| 70-74 | 360 | 0.46 | 0.52 | 0.57 | 0.61 | 0.69 | | 0.57 | 0.51 |
| 75-79 | 230 | 0.44 | 0.51 | 0.55 | 0.61 | 0.67 | | 0.55 | 0.50 |
| 80+ | 145 | 0.44 | 0.48 | 0.53 | 0.59 | 0.68 | | 0.54 | 0.48 |
| **aMFR (kg/kg)** | | | | | | | | | |
| 45-49 | 251 | 0.53 | 0.61 | 0.71 | 0.84 | 1.05 | | 0.73 | 1.29 |
| 50-54 | 274 | 0.48 | 0.57 | 0.67 | 0.77 | 0.97 | | 0.68 | 1.22 |
| 55-59 | 314 | 0.47 | 0.57 | 0.65 | 0.75 | 0.97 | | 0.68 | 1.25 |
| 60-64 | 318 | 0.44 | 0.55 | 0.63 | 0.74 | 0.93 | | 0.65 | 1.20 |
| 65-69 | 564 | 0.44 | 0.54 | 0.63 | 0.73 | 0.97 | | 0.66 | 1.19 |
| 70-74 | 360 | 0.44 | 0.54 | 0.62 | 0.72 | 1.01 | | 0.66 | 1.17 |
| 75-79 | 230 | 0.43 | 0.53 | 0.63 | 0.73 | 0.99 | | 0.67 | 1.16 |
| 80+ | 145 | 0.46 | 0.53 | 0.63 | 0.77 | 1.22 | | 0.69 | 1.20 |
| **tMFR (kg/kg)** | | | | | | | | | |
| 45-49 | 251 | 0.55 | 0.67 | 0.79 | 0.97 | 1.24 | 0.83 | | 0.65 |
| 50-54 | 274 | 0.51 | 0.62 | 0.75 | 0.87 | 1.16 | 0.77 | | 0.60 |
| 55-59 | 314 | 0.52 | 0.63 | 0.72 | 0.87 | 1.13 | 0.77 | | 0.61 |
| 60-64 | 318 | 0.49 | 0.60 | 0.72 | 0.83 | 1.13 | 0.74 | | 0.57 |
| 65-69 | 564 | 0.49 | 0.60 | 0.70 | 0.83 | 1.19 | 0.75 | | 0.59 |
| 70-74 | 360 | 0.47 | 0.60 | 0.71 | 0.84 | 1.22 | 0.76 | | 0.57 |
| 75-79 | 230 | 0.49 | 0.63 | 0.72 | 0.9 | 1.25 | 0.79 | | 0.59 |
| 80+ | 145 | 0.51 | 0.63 | 0.73 | 0.96 | 1.51 | 0.84 | | 0.61 |
| **Handgrip strength (kg)** | | | | | | | | | |
| 45-49 | 251 | 19 | 23 | 25 | 29 | 34 | | 26 | 22 |
| 50-54 | 274 | 17 | 21 | 25 | 29 | 34 | | 25 | 20 |
| 55-59 | 314 | 15 | 21 | 24 | 27 | 32 | | 24 | 20 |
| 60-64 | 318 | 15 | 20 | 24 | 27 | 32 | | 23 | 20 |
| 65-69 | 564 | 15 | 19 | 22 | 25 | 31 | | 22 | 19 |
| 70-74 | 360 | 13 | 19 | 22 | 24 | 29 | | 21 | 17 |
| 75-79 | 230 | 12 | 17 | 20 | 22 | 27 | | 19 | 16 |
| 80+ | 145 | 10 | 15 | 18 | 20 | 26 | | 17 | 14 |

ALM, appendicular lean mass; BMI, body mass index; aMFR, appendicular muscle-to-fat ratio;
tMFR, total muscle-to-fat ratio

For NHANES, all analyses were performed using the combined sample weights in accordance with the analytical guidelines. Five DXA imputation datasets were generated by NHANES, and calculations were performed separately for each imputed dataset according to the NHANES DXA technical documentation.

**Table 2. Age-specific normative values for metrics of muscle health among men**

A. **UK biobank**

|  | Observations | Percentiles | | | | | Mean | 20th |
| --- | --- | --- | --- | --- | --- | --- | --- | --- |
|  |  | **5th** | **25th** | **50th** | **75th** | **95th** |  |  |
| **ALM (kg)** | | | | | | | | |
| 45-49 | 323 | 21.5 | 24.3 | 26.7 | 29.8 | 34.9 | 27.4 | 23.8 |
| 50-54 | 1925 | 21.3 | 24.4 | 26.8 | 29.4 | 33.9 | 27.0 | 23.8 |
| 55-59 | 2670 | 20.9 | 24.1 | 26.3 | 28.8 | 32.9 | 26.6 | 23.5 |
| 60-64 | 3462 | 20.6 | 23.6 | 25.7 | 28.0 | 32.1 | 25.9 | 23.1 |
| 65-69 | 4309 | 20.2 | 22.9 | 25.1 | 27.3 | 31.0 | 25.2 | 22.4 |
| 70-74 | 3982 | 19.8 | 22.4 | 24.3 | 26.6 | 30.1 | 24.6 | 22.0 |
| 75-79 | 2148 | 19.1 | 21.6 | 23.5 | 25.7 | 28.9 | 23.7 | 21.1 |
| 80+ | 306 | 18.9 | 21.2 | 23.0 | 24.7 | 28.4 | 23.1 | 20.6 |
| **ALM, Height-adjusted (kg/m^2^)** | | | | | | | | |
| 45-49 | 323 | 7.00 | 7.79 | 8.51 | 9.35 | 10.61 | 8.63 | 7.63 |
| 50-54 | 1925 | 6.92 | 7.83 | 8.42 | 9.14 | 10.44 | 8.53 | 7.69 |
| 55-59 | 2670 | 6.91 | 7.78 | 8.38 | 9.03 | 10.25 | 8.44 | 7.64 |
| 60-64 | 3462 | 6.84 | 7.65 | 8.22 | 8.84 | 9.92 | 8.28 | 7.50 |
| 65-69 | 4309 | 6.81 | 7.52 | 8.07 | 8.67 | 9.70 | 8.14 | 7.41 |
| 70-74 | 3982 | 6.70 | 7.44 | 7.94 | 8.54 | 9.54 | 8.01 | 7.31 |
| 75-79 | 2148 | 6.55 | 7.25 | 7.77 | 8.35 | 9.28 | 7.82 | 7.14 |
| 80+ | 306 | 6.44 | 7.12 | 7.63 | 8.13 | 9.03 | 7.66 | 6.99 |
| **ALM, BMI-adjusted (kg/kg/m^2^)** | | | | | | | | |
| 45-49 | 323 | 0.83 | 0.93 | 1.03 | 1.09 | 1.24 | 1.02 | 0.91 |
| 50-54 | 1925 | 0.81 | 0.93 | 1.01 | 1.09 | 1.20 | 1.01 | 0.92 |
| 55-59 | 2670 | 0.80 | 0.91 | 0.98 | 1.06 | 1.19 | 0.99 | 0.89 |
| 60-64 | 3462 | 0.79 | 0.89 | 0.97 | 1.04 | 1.16 | 0.97 | 0.88 |
| 65-69 | 4309 | 0.77 | 0.87 | 0.95 | 1.02 | 1.14 | 0.95 | 0.86 |
| 70-74 | 3982 | 0.76 | 0.85 | 0.93 | 0.99 | 1.11 | 0.93 | 0.84 |
| 75-79 | 2148 | 0.74 | 0.84 | 0.90 | 0.98 | 1.09 | 0.91 | 0.82 |
| 80+ | 306 | 0.75 | 0.84 | 0.90 | 0.98 | 1.09 | 0.91 | 0.82 |
| **aMFR (kg/kg)** | | | | | | | | |
| 45-49 | 323 | 0.70 | 0.93 | 1.11 | 1.43 | 2.42 | 1.28 | 0.88 |
| 50-54 | 1925 | 0.69 | 0.91 | 1.11 | 1.37 | 2.10 | 1.22 | 0.87 |
| 55-59 | 2670 | 0.69 | 0.89 | 1.08 | 1.33 | 2.05 | 1.18 | 0.86 |
| 60-64 | 3462 | 0.68 | 0.89 | 1.07 | 1.30 | 1.94 | 1.16 | 0.86 |
| 65-69 | 4309 | 0.67 | 0.87 | 1.05 | 1.29 | 1.97 | 1.14 | 0.84 |
| 70-74 | 3982 | 0.67 | 0.86 | 1.02 | 1.25 | 1.83 | 1.11 | 0.82 |
| 75-79 | 2148 | 0.67 | 0.84 | 1.02 | 1.24 | 1.83 | 1.09 | 0.81 |
| 80+ | 306 | 0.71 | 0.88 | 1.02 | 1.26 | 1.88 | 1.12 | 0.85 |
| **tMFR (kg/kg)** | | | | | | | | |
| 45-49 | 323 | 1.47 | 1.95 | 2.37 | 3.04 | 5.44 | 2.73 | 1.85 |
| 50-54 | 1925 | 1.47 | 1.95 | 2.35 | 2.96 | 4.67 | 2.62 | 1.86 |
| 55-59 | 2670 | 1.46 | 1.91 | 2.32 | 2.90 | 4.57 | 2.55 | 1.83 |
| 60-64 | 3462 | 1.46 | 1.91 | 2.31 | 2.85 | 4.28 | 2.52 | 1.83 |
| 65-69 | 4309 | 1.43 | 1.89 | 2.29 | 2.86 | 4.39 | 2.52 | 1.81 |
| 70-74 | 3982 | 1.45 | 1.87 | 2.23 | 2.76 | 4.13 | 2.45 | 1.79 |
| 75-79 | 2148 | 1.47 | 1.87 | 2.27 | 2.77 | 4.18 | 2.45 | 1.79 |
| 80+ | 306 | 1.61 | 1.98 | 2.29 | 2.86 | 4.25 | 2.54 | 1.91 |
| **Handgrip strength (kg)** | | | | | | | | |
| 45-49 | 323 | 31 | 38 | 45 | 50 | 60 | 45 | 38 |
| 50-54 | 1925 | 30 | 38 | 44 | 50 | 60 | 44 | 36 |
| 55-59 | 2670 | 28 | 36 | 42 | 48 | 56 | 42 | 35 |
| 60-64 | 3462 | 28 | 35 | 40 | 46 | 55 | 41 | 34 |
| 65-69 | 4309 | 26 | 34 | 40 | 44 | 52 | 39 | 32 |
| 70-74 | 3982 | 24 | 32 | 37 | 42 | 50 | 37 | 30 |
| 75-79 | 2148 | 21 | 30 | 34 | 40 | 46 | 34 | 28 |
| 80+ | 306 | 20 | 28 | 32 | 38 | 43 | 32 | 26 |

B. **NHANES**

|  | Observations | Percentiles | | | | | Mean | 20th |
| --- | --- | --- | --- | --- | --- | --- | --- | --- |
|  |  | **5th** | **25th** | **50th** | **75th** | **95th** |  |  |
| **ALM (kg)** | | | | | | | | |
| 45-49 | 716 | 21.2 | 24.4 | 27.0 | 29.6 | 36.0 | 27.4 | 23.7 |
| 50-54 | 697 | 20.1 | 23.7 | 26.6 | 29.7 | 34.8 | 26.9 | 22.9 |
| 55-59 | 485 | 19.1 | 23.1 | 26.1 | 28.6 | 34.0 | 26.2 | 22.5 |
| 60-64 | 707 | 18.8 | 22.9 | 25.7 | 28.8 | 32.9 | 25.9 | 22.3 |
| 65-69 | 637 | 18.7 | 22.3 | 24.9 | 27.8 | 32.2 | 25.1 | 21.7 |
| 70-74 | 472 | 17.6 | 20.8 | 23.5 | 26.3 | 30.0 | 23.6 | 20.0 |
| 75-79 | 337 | 17.4 | 20.4 | 22.8 | 25.0 | 29.0 | 23.0 | 19.9 |
| 80+ | 465 | 15.9 | 18.9 | 20.9 | 23.1 | 26.4 | 21.1 | 18.4 |
| **ALM, Height-adjusted (kg/m^2^)** | | | | | | | | |
| 45-49 | 716 | 7.02 | 7.94 | 8.68 | 9.31 | 10.96 | 8.77 | 7.77 |
| 50-54 | 697 | 6.68 | 7.77 | 8.60 | 9.35 | 10.76 | 8.63 | 7.58 |
| 55-59 | 485 | 6.51 | 7.65 | 8.39 | 9.12 | 10.52 | 8.44 | 7.45 |
| 60-64 | 707 | 6.48 | 7.61 | 8.38 | 9.11 | 10.26 | 8.41 | 7.40 |
| 65-69 | 637 | 6.30 | 7.44 | 8.12 | 8.85 | 10.30 | 8.19 | 7.26 |
| 70-74 | 472 | 6.23 | 7.13 | 7.82 | 8.47 | 9.52 | 7.83 | 6.96 |
| 75-79 | 337 | 6.08 | 7.05 | 7.66 | 8.28 | 9.54 | 7.71 | 6.85 |
| 80+ | 465 | 5.82 | 6.58 | 7.23 | 7.80 | 8.73 | 7.24 | 6.45 |
| **ALM, BMI-adjusted (kg/kg/m^2^)** | | | | | | | | |
| 45-49 | 716 | 0.76 | 0.88 | 0.96 | 1.04 | 1.19 | 0.97 | 0.87 |
| 50-54 | 697 | 0.75 | 0.86 | 0.94 | 1.02 | 1.16 | 0.94 | 0.84 |
| 55-59 | 485 | 0.74 | 0.84 | 0.91 | 0.99 | 1.12 | 0.92 | 0.83 |
| 60-64 | 707 | 0.72 | 0.81 | 0.90 | 0.98 | 1.09 | 0.90 | 0.80 |
| 65-69 | 637 | 0.69 | 0.79 | 0.87 | 0.95 | 1.07 | 0.87 | 0.78 |
| 70-74 | 472 | 0.67 | 0.76 | 0.85 | 0.93 | 1.04 | 0.85 | 0.75 |
| 75-79 | 337 | 0.68 | 0.77 | 0.84 | 0.91 | 1.01 | 0.84 | 0.75 |
| 80+ | 465 | 0.64 | 0.74 | 0.81 | 0.88 | 1.00 | 0.81 | 0.72 |
| **aMFR (kg/kg)** | | | | | | | | |
| 45-49 | 716 | 0.68 | 0.90 | 1.10 | 1.31 | 1.83 | 1.15 | 0.86 |
| 50-54 | 697 | 0.67 | 0.87 | 1.02 | 1.25 | 1.77 | 1.10 | 0.82 |
| 55-59 | 485 | 0.64 | 0.83 | 0.98 | 1.18 | 1.57 | 1.04 | 0.80 |
| 60-64 | 707 | 0.64 | 0.80 | 0.96 | 1.15 | 1.51 | 1.01 | 0.78 |
| 65-69 | 637 | 0.61 | 0.76 | 0.91 | 1.09 | 1.41 | 0.95 | 0.72 |
| 70-74 | 472 | 0.58 | 0.77 | 0.92 | 1.10 | 1.48 | 0.96 | 0.73 |
| 75-79 | 337 | 0.61 | 0.77 | 0.90 | 1.04 | 1.38 | 0.94 | 0.74 |
| 80+ | 465 | 0.61 | 0.77 | 0.90 | 1.07 | 1.43 | 0.94 | 0.74 |
| **tMFR (kg/kg)** | | | | | | | | |
| 45-49 | 716 | 1.56 | 2.03 | 2.44 | 2.90 | 4.06 | 2.58 | 1.94 |
| 50-54 | 697 | 1.55 | 2.00 | 2.31 | 2.80 | 3.98 | 2.48 | 1.90 |
| 55-59 | 485 | 1.50 | 1.91 | 2.23 | 2.64 | 3.64 | 2.37 | 1.85 |
| 60-64 | 707 | 1.50 | 1.86 | 2.22 | 2.61 | 3.46 | 2.31 | 1.80 |
| 65-69 | 637 | 1.44 | 1.78 | 2.12 | 2.50 | 3.25 | 2.20 | 1.72 |
| 70-74 | 472 | 1.39 | 1.81 | 2.13 | 2.54 | 3.44 | 2.25 | 1.73 |
| 75-79 | 337 | 1.50 | 1.81 | 2.10 | 2.46 | 3.09 | 2.18 | 1.73 |
| 80+ | 465 | 1.50 | 1.84 | 2.13 | 2.51 | 3.36 | 2.24 | 1.76 |
| **Handgrip strength (kg)** | | | | | | | | |
| 45-49 | 377 | 35 | 43 | 48 | 52 | 61 | 48 | 42 |
| 50-54 | 392 | 29 | 40 | 46 | 51 | 59 | 45 | 38 |
| 55-59 | 370 | 33 | 39 | 45 | 49 | 56 | 44 | 38 |
| 60-64 | 443 | 31 | 39 | 44 | 48 | 57 | 43 | 37 |
| 65-69 | 311 | 28 | 35 | 42 | 46 | 53 | 41 | 34 |
| 70-74 | 244 | 26 | 33 | 39 | 43 | 50 | 38 | 32 |
| 75-79 | 175 | 26 | 30 | 36 | 41 | 48 | 36 | 29 |
| 80+ | 266 | 17 | 26 | 32 | 37 | 43 | 31 | 26 |

C. **NAHSIT**

|  | Observations | Percentiles | | | | | Mean | 20th |
| --- | --- | --- | --- | --- | --- | --- | --- | --- |
|  |  | **5th** | **25th** | **50th** | **75th** | **95th** |  |  |
| **ALM (kg)** | | | | | | | | |
| 45-49 | 181 | 17.5 | 20.5 | 22.7 | 25.7 | 29.3 | 23.1 | 19.7 |
| 50-54 | 238 | 17.1 | 20.0 | 22.3 | 24.3 | 27.6 | 22.3 | 19.5 |
| 55-59 | 319 | 16.7 | 20.0 | 21.7 | 23.8 | 27.3 | 22.0 | 19.5 |
| 60-64 | 327 | 16.5 | 19.2 | 21.0 | 22.9 | 25.9 | 21.0 | 18.6 |
| 65-69 | 579 | 16.1 | 18.6 | 20.5 | 22.5 | 25.3 | 20.6 | 18.1 |
| 70-74 | 360 | 15.1 | 17.4 | 19.8 | 21.6 | 24.5 | 19.7 | 17.1 |
| 75-79 | 264 | 14.4 | 17.2 | 18.7 | 20.2 | 23.1 | 18.8 | 16.5 |
| 80+ | 235 | 13.5 | 16.1 | 17.8 | 19.5 | 22.6 | 17.9 | 15.5 |
| **ALM, Height-adjusted (kg/m^2^)** | | | | | | | | |
| 45-49 | 181 | 6.33 | 7.23 | 7.90 | 8.78 | 9.75 | 8.03 | 7.12 |
| 50-54 | 238 | 6.13 | 7.28 | 7.82 | 8.38 | 9.57 | 7.83 | 7.05 |
| 55-59 | 319 | 6.36 | 7.24 | 7.78 | 8.42 | 9.41 | 7.84 | 7.12 |
| 60-64 | 327 | 6.33 | 7.07 | 7.60 | 8.20 | 9.07 | 7.63 | 6.91 |
| 65-69 | 579 | 6.18 | 7.00 | 7.57 | 8.16 | 9.11 | 7.58 | 6.88 |
| 70-74 | 360 | 5.88 | 6.76 | 7.31 | 7.87 | 8.85 | 7.32 | 6.65 |
| 75-79 | 264 | 5.76 | 6.49 | 7.03 | 7.62 | 8.60 | 7.09 | 6.33 |
| 80+ | 235 | 5.42 | 6.16 | 6.78 | 7.35 | 8.50 | 6.82 | 6.05 |
| **ALM, BMI-adjusted (kg/kg/m^2^)** | | | | | | | | |
| 45-49 | 181 | 0.75 | 0.85 | 0.91 | 0.96 | 1.06 | 0.91 | 0.83 |
| 50-54 | 238 | 0.73 | 0.81 | 0.89 | 0.96 | 1.07 | 0.89 | 0.80 |
| 55-59 | 319 | 0.69 | 0.8 | 0.87 | 0.93 | 1.04 | 0.87 | 0.79 |
| 60-64 | 327 | 0.69 | 0.78 | 0.84 | 0.9 | 1.01 | 0.84 | 0.76 |
| 65-69 | 579 | 0.67 | 0.75 | 0.81 | 0.87 | 0.99 | 0.82 | 0.74 |
| 70-74 | 360 | 0.65 | 0.73 | 0.79 | 0.86 | 0.98 | 0.80 | 0.72 |
| 75-79 | 264 | 0.64 | 0.72 | 0.77 | 0.83 | 0.94 | 0.77 | 0.70 |
| 80+ | 235 | 0.62 | 0.69 | 0.75 | 0.82 | 0.92 | 0.76 | 0.68 |
| **aMFR (kg/kg)** | | | | | | | | |
| 45-49 | 181 | 0.77 | 0.96 | 1.13 | 1.32 | 2.13 | 1.21 | 2.27 |
| 50-54 | 238 | 0.74 | 0.93 | 1.10 | 1.34 | 2.25 | 1.21 | 2.32 |
| 55-59 | 319 | 0.71 | 0.93 | 1.08 | 1.29 | 1.81 | 1.17 | 2.25 |
| 60-64 | 327 | 0.73 | 0.91 | 1.04 | 1.29 | 2.02 | 1.17 | 2.26 |
| 65-69 | 579 | 0.69 | 0.86 | 0.99 | 1.18 | 1.74 | 1.08 | 2.12 |
| 70-74 | 360 | 0.67 | 0.84 | 0.99 | 1.19 | 1.68 | 1.07 | 2.12 |
| 75-79 | 264 | 0.67 | 0.81 | 0.95 | 1.19 | 1.92 | 1.12 | 2.01 |
| 80+ | 235 | 0.66 | 0.79 | 0.91 | 1.13 | 2.00 | 1.10 | 1.91 |
| **tMFR (kg/kg)** | | | | | | | | |
| 45-49 | 181 | 0.75 | 0.98 | 1.15 | 1.35 | 2.11 | 1.24 | 0.92 |
| 50-54 | 238 | 0.74 | 0.96 | 1.15 | 1.40 | 2.44 | 1.27 | 0.90 |
| 55-59 | 319 | 0.74 | 0.95 | 1.12 | 1.39 | 2.03 | 1.24 | 0.90 |
| 60-64 | 327 | 0.77 | 0.95 | 1.11 | 1.38 | 2.25 | 1.25 | 0.91 |
| 65-69 | 579 | 0.73 | 0.89 | 1.05 | 1.29 | 1.95 | 1.16 | 0.86 |
| 70-74 | 360 | 0.71 | 0.91 | 1.07 | 1.34 | 2.00 | 1.18 | 0.86 |
| 75-79 | 264 | 0.70 | 0.88 | 1.05 | 1.34 | 2.39 | 1.27 | 0.84 |
| 80+ | 235 | 0.70 | 0.86 | 1.03 | 1.30 | 2.37 | 1.27 | 0.83 |
| **Handgrip strength (kg)** | | | | | | | | |
| 45-49 | 181 | 33 | 40 | 45 | 51 | 61 | 45 | 38 |
| 50-54 | 238 | 31 | 39 | 43 | 48 | 57 | 43 | 37 |
| 55-59 | 319 | 29 | 36 | 42 | 46 | 56 | 42 | 35 |
| 60-64 | 327 | 29 | 35 | 40 | 44 | 51 | 40 | 34 |
| 65-69 | 579 | 24 | 33 | 38 | 42 | 50 | 37 | 31 |
| 70-74 | 360 | 23 | 30 | 34 | 39 | 46 | 35 | 29 |
| 75-79 | 264 | 23 | 28 | 32 | 36 | 44 | 32 | 27 |
| 80+ | 235 | 16 | 24 | 29 | 34 | 41 | 28 | 23 |

ALM, appendicular lean mass; BMI, body mass index; aMFR, appendicular muscle-to-fat ratio;
tMFR, total muscle-to-fat ratio

For NHANES, all analyses were performed using the combined sample weights in accordance with the analytical guidelines. Five DXA imputation datasets were generated by NHANES, and calculations were performed separately for each imputed dataset according to the NHANES DXA technical documentation.

**Table 3. Age-specific normative values for adiposity measures among women**

A. **UK biobank**

|  | Observations | Percentiles | | | | | Mean | 20th |
| --- | --- | --- | --- | --- | --- | --- | --- | --- |
|  |  | **5th** | **25th** | **50th** | **75th** | **95th** |  |  |
| **Fat mass (kg)** | | | | | | | | |
| 45-49 | 432 | 13.1 | 18.8 | 24.0 | 32.5 | 43.9 | 26.3 | 17.3 |
| 50-54 | 2402 | 13.5 | 19.5 | 25.0 | 32.8 | 48.1 | 27.2 | 18.3 |
| 55-59 | 3519 | 13.7 | 20.0 | 25.3 | 32.6 | 47.1 | 27.1 | 18.8 |
| 60-64 | 4156 | 13.3 | 20.0 | 25.6 | 32.6 | 45.4 | 27.1 | 18.7 |
| 65-69 | 4467 | 13.7 | 19.7 | 25.0 | 31.3 | 43.8 | 26.3 | 18.5 |
| 70-74 | 3569 | 13.5 | 20.1 | 25.0 | 31.3 | 41.9 | 26.1 | 19.0 |
| 75-79 | 1555 | 13.1 | 19.6 | 24.4 | 29.9 | 40.6 | 25.3 | 18.3 |
| 80+ | 211 | 11.3 | 17.3 | 22.5 | 28.6 | 37.1 | 23.6 | 16.6 |
| **Fat percentage (%)** | | | | | | | | |
| 45-49 | 432 | 24.0 | 31.2 | 36.3 | 41.45 | 47.8 | 36.2 | 29.7 |
| 50-54 | 2402 | 24.2 | 31.8 | 37.1 | 42.6 | 49.5 | 37.1 | 30.4 |
| 55-59 | 3519 | 24.9 | 32.7 | 37.8 | 43.0 | 49.6 | 37.7 | 31.4 |
| 60-64 | 4156 | 24.7 | 33.2 | 38.6 | 43.3 | 49.6 | 38.0 | 31.9 |
| 65-69 | 4467 | 25.8 | 33.1 | 38.2 | 42.9 | 49.0 | 37.9 | 31.9 |
| 70-74 | 3569 | 25.8 | 33.8 | 38.6 | 42.9 | 48.8 | 38.1 | 32.5 |
| 75-79 | 1555 | 25.5 | 33.4 | 38.0 | 42.4 | 47.8 | 37.6 | 31.8 |
| 80+ | 211 | 22.3 | 30.9 | 37.2 | 41.8 | 48.1 | 36.1 | 29.3 |

B. **NHANES**

|  | Observations | Percentiles | | | | | Mean | 20th |
| --- | --- | --- | --- | --- | --- | --- | --- | --- |
|  |  | **5th** | **25th** | **50th** | **75th** | **95th** |  |  |
| **Fat mass (kg)** | | | | | | | | |
| 45-49 | 747 | 15.1 | 22.0 | 29.9 | 40.7 | 58.6 | 32.6 | 20.9 |
| 50-54 | 706 | 16.1 | 23.5 | 30.8 | 38.1 | 56.2 | 32.6 | 22.2 |
| 55-59 | 487 | 17.0 | 24.7 | 32.2 | 41.7 | 60.6 | 34.5 | 22.8 |
| 60-64 | 780 | 18.3 | 25.5 | 32.5 | 39.5 | 57.3 | 33.9 | 24.3 |
| 65-69 | 628 | 17.9 | 25.4 | 32.1 | 39.7 | 55.3 | 33.6 | 24.0 |
| 70-74 | 414 | 16.3 | 23.8 | 29.9 | 36.4 | 48.1 | 30.8 | 22.3 |
| 75-79 | 310 | 16.9 | 24.9 | 29.2 | 34.7 | 47.4 | 30.5 | 24.0 |
| 80+ | 569 | 14.0 | 20.8 | 25.9 | 31.0 | 42.1 | 26.4 | 20.0 |
| **Fat percentage (%)** | | | | | | | | |
| 45-49 | 747 | 27.3 | 35.4 | 40.5 | 45.1 | 50.7 | 40.1 | 34.1 |
| 50-54 | 706 | 29.7 | 37.0 | 41.5 | 45.2 | 50.5 | 40.9 | 35.8 |
| 55-59 | 487 | 30.0 | 38.3 | 42.9 | 46.3 | 51.9 | 42.3 | 37.3 |
| 60-64 | 780 | 32.2 | 38.6 | 43.0 | 46.6 | 50.8 | 42.5 | 37.8 |
| 65-69 | 628 | 32.8 | 39.1 | 43.0 | 46.5 | 51.7 | 42.8 | 38.2 |
| 70-74 | 414 | 31.2 | 38.5 | 42.7 | 46.4 | 50.5 | 42.1 | 37.0 |
| 75-79 | 310 | 31.4 | 39.2 | 42.7 | 45.4 | 50.0 | 42.2 | 38.3 |
| 80+ | 569 | 29.7 | 36.9 | 41.0 | 44.4 | 48.9 | 40.4 | 35.2 |

C. **NAHSIT**

|  | Observations | Percentiles | | | | | Mean | 20th |
| --- | --- | --- | --- | --- | --- | --- | --- | --- |
|  |  | **5th** | **25th** | **50th** | **75th** | **95th** |  |  |
| **Fat mass (kg)** | | | | | | | | |
| 45-49 | 251 | 13.0 | 17.6 | 21.4 | 26.3 | 33.9 | 22.3 | 16.7 |
| 50-54 | 274 | 14.3 | 18.7 | 22.6 | 27.6 | 35.7 | 23.6 | 17.3 |
| 55-59 | 314 | 14.3 | 18.9 | 22.5 | 26.9 | 33.9 | 23.2 | 18.4 |
| 60-64 | 318 | 13.5 | 19.5 | 22.5 | 27.7 | 36.5 | 23.8 | 18.7 |
| 65-69 | 564 | 13.5 | 19.2 | 22.9 | 27.5 | 35.3 | 23.5 | 18.1 |
| 70-74 | 360 | 13.0 | 19.0 | 23.0 | 27.4 | 35.7 | 23.5 | 18.0 |
| 75-79 | 230 | 12.2 | 17.2 | 21.6 | 26.4 | 35.0 | 22.3 | 16.2 |
| 80+ | 145 | 10.0 | 15.9 | 20.9 | 24.6 | 30.7 | 20.7 | 14.9 |
| **Fat percentage (%)** | | | | | | | | |
| 45-49 | 251 | 27.5 | 32.5 | 36.4 | 40.2 | 45.0 | 36.4 | 31.6 |
| 50-54 | 274 | 28.9 | 34.3 | 37.8 | 42.1 | 46.3 | 38.0 | 33.6 |
| 55-59 | 314 | 29.1 | 34.8 | 38.7 | 41.9 | 46.5 | 38.2 | 34.0 |
| 60-64 | 318 | 29.8 | 35.6 | 39.1 | 42.9 | 47.7 | 39.1 | 34.9 |
| 65-69 | 564 | 28.6 | 35.8 | 39.5 | 42.8 | 47.7 | 39.1 | 34.6 |
| 70-74 | 360 | 28.1 | 35.8 | 39.8 | 43.0 | 48.7 | 39.1 | 34.4 |
| 75-79 | 230 | 28.9 | 34.4 | 38.9 | 42.9 | 47.9 | 38.6 | 33.6 |
| 80+ | 145 | 23.8 | 33.6 | 39.1 | 42.6 | 47.4 | 37.7 | 32.8 |

For NHANES, all analyses were performed using the combined sample weights in accordance with the analytical guidelines. Five DXA imputation datasets were generated by NHANES, and calculations were performed separately for each imputed dataset according to the NHANES DXA technical documentation.

**Table 4. Age-specific normative values for adiposity measures among men**

A. **UK biobank**

|  | Observations | Percentiles | | | | | Mean (SD) | 20th |
| --- | --- | --- | --- | --- | --- | --- | --- | --- |
|  |  | **5th** | **25th** | **50th** | **75th** | **95th** |  |  |
| **Fat mass (kg)** | | | | | | | | |
| 45-49 | 323 | 9.9 | 18.7 | 23.5 | 29.7 | 42.3 | 25.1 | 17.5 |
| 50-54 | 1925 | 12.0 | 18.7 | 24.0 | 29.5 | 42.3 | 25.1 | 17.6 |
| 55-59 | 2670 | 12.2 | 19.0 | 24.1 | 30.0 | 42.1 | 25.3 | 17.9 |
| 60-64 | 3462 | 12.4 | 18.8 | 23.8 | 29.7 | 40.8 | 25.0 | 17.8 |
| 65-69 | 4309 | 12.2 | 18.7 | 23.7 | 29.3 | 40.9 | 24.7 | 17.5 |
| 70-74 | 3982 | 12.6 | 19.1 | 23.8 | 29.3 | 39.2 | 24.6 | 17.7 |
| 75-79 | 2148 | 12.4 | 18.5 | 22.9 | 28.3 | 37.8 | 23.8 | 17.5 |
| 80+ | 306 | 11.7 | 17.8 | 22.0 | 26.6 | 34.2 | 22.4 | 16.4 |
| **Fat percentage (%)** | | | | | | | | |
| 45-49 | 323 | 14.9 | 23.8 | 28.7 | 32.6 | 39.2 | 28.1 | 22.3 |
| 50-54 | 1925 | 16.8 | 24.3 | 28.8 | 32.6 | 39.3 | 28.5 | 22.9 |
| 55-59 | 2670 | 17.2 | 24.7 | 29.0 | 33.2 | 39.4 | 28.8 | 23.7 |
| 60-64 | 3462 | 18.1 | 24.9 | 29.0 | 33.1 | 39.3 | 29.0 | 23.8 |
| 65-69 | 4309 | 17.7 | 24.8 | 29.2 | 33.4 | 39.8 | 29.1 | 23.7 |
| 70-74 | 3982 | 18.7 | 25.5 | 29.8 | 33.6 | 39.5 | 29.5 | 24.5 |
| 75-79 | 2148 | 18.5 | 25.5 | 29.4 | 33.6 | 39.3 | 29.4 | 24.5 |
| 80+ | 306 | 18.2 | 25.0 | 29.3 | 32.2 | 37.0 | 28.5 | 23.7 |

B. **NHANES**

|  | Observations | Percentiles | | | | | Mean | 20th |
| --- | --- | --- | --- | --- | --- | --- | --- | --- |
|  |  | **5th** | **25th** | **50th** | **75th** | **95th** |  |  |
| **Fat mass (kg)** | | | | | | | | |
| 45-49 | 716 | 12.9 | 19.6 | 24.7 | 31.1 | 46.5 | 26.4 | 18.2 |
| 50-54 | 697 | 13.4 | 20.4 | 25.5 | 32.3 | 46.5 | 27.1 | 18.9 |
| 55-59 | 485 | 13.1 | 20.6 | 26.3 | 33.0 | 47.4 | 27.5 | 19.8 |
| 60-64 | 707 | 14.5 | 21.3 | 26.5 | 33.1 | 45.7 | 27.9 | 20.2 |
| 65-69 | 637 | 15.2 | 21.8 | 26.9 | 34.3 | 47.1 | 28.5 | 20.9 |
| 70-74 | 472 | 12.8 | 20.0 | 25.7 | 32.5 | 43.3 | 26.8 | 19.0 |
| 75-79 | 337 | 15.2 | 20.6 | 25.6 | 30.9 | 40.3 | 26.1 | 19.8 |
| 80+ | 465 | 13.1 | 19.2 | 23.0 | 27.9 | 37.1 | 23.9 | 18.1 |
| **Fat percentage (%)** | | | | | | | | |
| 45-49 | 716 | 18.9 | 24.7 | 28.2 | 31.9 | 38.0 | 28.3 | 23.6 |
| 50-54 | 697 | 19.2 | 25.4 | 29.2 | 32.3 | 38.2 | 29.0 | 24.4 |
| 55-59 | 485 | 20.6 | 26.5 | 29.9 | 33.4 | 39.0 | 29.8 | 25.4 |
| 60-64 | 707 | 21.5 | 26.6 | 30.0 | 34.0 | 39.0 | 30.2 | 25.8 |
| 65-69 | 637 | 22.6 | 27.5 | 31.0 | 34.8 | 39.9 | 31.2 | 26.9 |
| 70-74 | 472 | 21.5 | 27.2 | 30.8 | 34.5 | 40.6 | 30.9 | 26.0 |
| 75-79 | 337 | 23.3 | 27.9 | 31.2 | 34.6 | 38.8 | 31.3 | 27.1 |
| 80+ | 465 | 22.0 | 27.4 | 30.9 | 34.0 | 38.8 | 30.8 | 26.7 |

C. **NAHSIT**

|  | Observations | Percentiles | | | | | Mean | 20th |
| --- | --- | --- | --- | --- | --- | --- | --- | --- |
|  |  | **5th** | **25th** | **50th** | **75th** | **95th** |  |  |
| **Fat mass (kg)** | | | | | | | | |
| 45-49 | 181 | 10.9 | 16.5 | 20.0 | 24.7 | 33.5 | 21.0 | 15.6 |
| 50-54 | 238 | 9.4 | 15.8 | 19.7 | 24.6 | 34.4 | 20.5 | 15.0 |
| 55-59 | 319 | 10.8 | 16.5 | 20.0 | 24.3 | 32.7 | 20.8 | 15.8 |
| 60-64 | 327 | 9.6 | 16.0 | 19.9 | 23.9 | 29.5 | 19.9 | 14.4 |
| 65-69 | 579 | 11.0 | 16.8 | 20.6 | 24.7 | 30.6 | 20.8 | 15.7 |
| 70-74 | 360 | 10.4 | 15.6 | 19.7 | 24.4 | 31.6 | 20.2 | 15.0 |
| 75-79 | 264 | 9.2 | 14.8 | 19.7 | 23.5 | 30.2 | 19.5 | 13.9 |
| 80+ | 235 | 7.8 | 14.9 | 19.7 | 22.9 | 28.7 | 19.0 | 13.9 |
| **Fat percentage (%)** | | | | | | | | |
| 45-49 | 181 | 17.1 | 25.0 | 27.9 | 31.3 | 36.5 | 27.8 | 24.0 |
| 50-54 | 238 | 15.8 | 24.5 | 28.0 | 31.9 | 37.8 | 27.8 | 23.1 |
| 55-59 | 319 | 18.3 | 24.8 | 28.7 | 32.0 | 37.3 | 28.3 | 23.7 |
| 60-64 | 327 | 16.8 | 25.1 | 28.8 | 32.2 | 36.7 | 28.2 | 23.3 |
| 65-69 | 579 | 19.0 | 26.3 | 29.9 | 33.2 | 37.6 | 29.5 | 24.8 |
| 70-74 | 360 | 19.4 | 25.6 | 29.7 | 33.3 | 38.6 | 29.4 | 24.8 |
| 75-79 | 264 | 16.6 | 25.5 | 30.4 | 34.0 | 38.4 | 29.3 | 24.0 |
| 80+ | 235 | 16.6 | 26.6 | 30.8 | 34.5 | 38.4 | 29.6 | 24.3 |

For NHANES, all analyses were performed using the combined sample weights in accordance with the analytical guidelines. Five DXA imputation datasets were generated by NHANES, and calculations were performed separately for each imputed dataset according to the NHANES DXA technical documentation.

**Table 5. Age-specific normative values for muscle-related biomarkers among women**

A. **UK biobank**

|  | Observations | Percentiles | | | | | Mean | 20th |
| --- | --- | --- | --- | --- | --- | --- | --- | --- |
|  |  | **5th** | **25th** | **50th** | **75th** | **95th** |  |  |
| **CRP (mg/dL)** | | | | | | | | |
| 45-49 | 762 | 0.02 | 0.04 | 0.09 | 0.22 | 0.78 | 0.21 | 0.04 |
| 50-54 | 1037 | 0.02 | 0.05 | 0.10 | 0.23 | 0.72 | 0.22 | 0.04 |
| 55-59 | 1561 | 0.03 | 0.06 | 0.11 | 0.26 | 0.84 | 0.23 | 0.05 |
| 60-64 | 2234 | 0.03 | 0.06 | 0.13 | 0.26 | 0.79 | 0.24 | 0.05 |
| 65-69 | 2240 | 0.03 | 0.07 | 0.14 | 0.26 | 0.78 | 0.25 | 0.06 |
| 70-74 | 984 | 0.03 | 0.08 | 0.14 | 0.26 | 0.86 | 0.25 | 0.07 |
| 75-79 | 28 | 0.03 | 0.07 | 0.15 | 0.52 | 2.06 | 0.35 | 0.06 |
| 80+ | NA | NA | NA | NA | NA | NA | NA | NA |
| **Hb1AC (%)** | | | | | | | | |
| 45-49 | 636 | 4.7 | 5.0 | 5.2 | 5.4 | 5.8 | 5.3 | 5.0 |
| 50-54 | 840 | 4.8 | 5.1 | 5.3 | 5.5 | 5.9 | 5.4 | 5.1 |
| 55-59 | 1262 | 4.9 | 5.2 | 5.4 | 5.6 | 6.1 | 5.4 | 5.2 |
| 60-64 | 1786 | 5.0 | 5.2 | 5.4 | 5.7 | 6.2 | 5.5 | 5.2 |
| 65-69 | 1912 | 5.0 | 5.3 | 5.5 | 5.7 | 6.2 | 5.5 | 5.2 |
| 70-74 | 831 | 5.1 | 5.4 | 5.6 | 5.8 | 6.3 | 5.6 | 5.3 |
| 75-79 | 26 | 5.2 | 5.3 | 5.5 | 5.8 | 6.5 | 5.7 | 5.3 |
| 80+ | NA | NA | NA | NA | NA | NA | NA | NA |
| **Glucose (mg/dL)** | | | | | | | | |
| 45-49 | 649 | 72 | 81 | 86 | 91 | 103 | 87 | 80 |
| 50-54 | 900 | 73 | 82 | 87 | 92 | 103 | 88 | 80 |
| 55-59 | 1365 | 74 | 83 | 88 | 93 | 105 | 89 | 82 |
| 60-64 | 1941 | 75 | 83 | 89 | 94 | 110 | 91 | 82 |
| 65-69 | 1954 | 76 | 84 | 90 | 96 | 104 | 92 | 83 |
| 70-74 | 872 | 76 | 85 | 91 | 97 | 116 | 93 | 84 |
| 75-79 | 22 | 82 | 85 | 94 | 100 | 138 | 99 | 85 |
| 80+ | NA | NA | NA | NA | NA | NA | NA | NA |
| **Total cholesterol (mg/dL)** | | | | | | | | |
| 45-49 | 764 | 156 | 187 | 209 | 234 | 282 | 213 | 181 |
| 50-54 | 1039 | 164 | 200 | 225 | 254 | 283 | 228 | 193 |
| 55-59 | 1563 | 165 | 206 | 232 | 264 | 307 | 234 | 198 |
| 60-64 | 2240 | 169 | 210 | 238 | 266 | 309 | 239 | 203 |
| 65-69 | 2245 | 165 | 205 | 235 | 264 | 313 | 236 | 198 |
| 70-74 | 986 | 156 | 196 | 230 | 261 | 311 | 231 | 189 |
| 75-79 | 28 | 132 | 176 | 195 | 238 | 321 | 205 | 164 |
| 80+ | NA | NA | NA | NA | NA | NA | NA | NA |
| **HDL (mg/dL)** | | | | | | | | |
| 45-49 | 652 | 41 | 52 | 61 | 71 | 88 | 62 | 50 |
| 50-54 | 901 | 43 | 55 | 63 | 73 | 90 | 65 | 53 |
| 55-59 | 1365 | 43 | 55 | 64 | 75 | 90 | 65 | 52 |
| 60-64 | 1944 | 44 | 56 | 65 | 75 | 93 | 66 | 53 |
| 65-69 | 1952 | 44 | 55 | 64 | 75 | 94 | 66 | 53 |
| 70-74 | 872 | 42 | 54 | 65 | 74 | 94 | 66 | 52 |
| 75-79 | 22 | 50 | 55 | 61 | 69 | 83 | 63 | 54 |
| 80+ | NA | NA | NA | NA | NA | NA | NA | NA |
| **LDL (mg/dL)** | | | | | | | | |
| 45-49 | 761 | 89 | 109 | 127 | 145 | 186 | 130 | 105 |
| 50-54 | 1037 | 90 | 118 | 137 | 161 | 196 | 140 | 113 |
| 55-59 | 1562 | 93 | 122 | 143 | 167 | 204 | 145 | 117 |
| 60-64 | 2234 | 95 | 124 | 146 | 169 | 204 | 147 | 119 |
| 65-69 | 2239 | 90 | 120 | 144 | 168 | 205 | 145 | 115 |
| 70-74 | 982 | 87 | 114 | 141 | 163 | 205 | 141 | 108 |
| 75-79 | 28 | 71 | 90 | 110 | 141 | 202 | 118 | 89 |
| 80+ | NA | NA | NA | NA | NA | NA | NA | NA |
| **Triglycerides (mg/dL)** | | | | | | | | |
| 45-49 | 761 | 52 | 72 | 99 | 139 | 245 | 117 | 68 |
| 50-54 | 1037 | 56 | 80 | 108 | 151 | 250 | 125 | 75 |
| 55-59 | 1562 | 61 | 88 | 119 | 170 | 266 | 137 | 83 |
| 60-64 | 2235 | 66 | 95 | 126 | 173 | 285 | 144 | 88 |
| 65-69 | 2242 | 68 | 97 | 127 | 173 | 278 | 145 | 91 |
| 70-74 | 985 | 68 | 95 | 127 | 173 | 271 | 143 | 89 |
| 75-79 | 28 | 70 | 109 | 125 | 154 | 181 | 128 | 97 |
| 80+ | NA | NA | NA | NA | NA | NA | NA | NA |

B. **NHANES**

|  | Observations | Percentiles | | | | | Mean | 20th |
| --- | --- | --- | --- | --- | --- | --- | --- | --- |
|  |  | **5th** | **25th** | **50th** | **75th** | **95th** |  |  |
| **CRP (mg/dL)** | | | | | | | | |
| 45-49 | 1231 | 0.02 | 0.08 | 0.25 | 0.64 | 1.61 | 0.49 | 0.06 |
| 50-54 | 1129 | 0.02 | 0.08 | 0.24 | 0.58 | 1.83 | 0.50 | 0.06 |
| 55-59 | 860 | 0.02 | 0.10 | 0.25 | 0.57 | 1.75 | 0.49 | 0.08 |
| 60-64 | 1301 | 0.04 | 0.13 | 0.29 | 0.63 | 1.95 | 0.57 | 0.10 |
| 65-69 | 958 | 0.03 | 0.12 | 0.27 | 0.62 | 1.57 | 0.50 | 0.09 |
| 70-74 | 921 | 0.03 | 0.13 | 0.28 | 0.56 | 1.68 | 0.51 | 0.10 |
| 75-79 | 663 | 0.04 | 0.11 | 0.26 | 0.51 | 1.79 | 0.52 | 0.09 |
| 80+ | 1123 | 0.03 | 0.10 | 0.23 | 0.46 | 1.77 | 0.51 | 0.08 |
| **Hb1AC (%)** | | | | | | | | |
| 45-49 | 2125 | 4.8 | 5.1 | 5.3 | 5.6 | 6.5 | 5.5 | 5.0 |
| 50-54 | 2071 | 4.9 | 5.2 | 5.6 | 5.8 | 7.3 | 5.7 | 5.2 |
| 55-59 | 1734 | 5.0 | 5.3 | 5.5 | 5.8 | 7.6 | 5.8 | 5.2 |
| 60-64 | 2359 | 5.0 | 5.3 | 5.6 | 5.9 | 7.5 | 5.9 | 5.2 |
| 65-69 | 1732 | 5.0 | 5.4 | 5.6 | 5.9 | 7.5 | 5.8 | 5.3 |
| 70-74 | 1541 | 5.0 | 5.4 | 5.6 | 6.0 | 7.5 | 5.9 | 5.3 |
| 75-79 | 1085 | 5.0 | 5.4 | 5.6 | 6.0 | 7.5 | 5.9 | 5.3 |
| 80+ | 1826 | 5.0 | 5.4 | 5.7 | 6.0 | 7.3 | 5.9 | 5.3 |
| **Glucose (mg/dL)** | | | | | | | | |
| 45-49 | 1013 | 83 | 90 | 97 | 105 | 131 | 102 | 89 |
| 50-54 | 996 | 83 | 92 | 98 | 106 | 141 | 104 | 90 |
| 55-59 | 871 | 84 | 94 | 100 | 109 | 181 | 110 | 92 |
| 60-64 | 1172 | 85 | 94 | 101 | 113 | 168 | 110 | 93 |
| 65-69 | 857 | 85 | 95 | 104 | 118 | 171 | 113 | 93 |
| 70-74 | 756 | 83 | 95 | 105 | 117 | 165 | 112 | 93 |
| 75-79 | 540 | 86 | 96 | 104 | 117 | 172 | 113 | 94 |
| 80+ | 893 | 85 | 96 | 103 | 117 | 164 | 111 | 94 |
| **Total cholesterol (mg/dL)** | | | | | | | | |
| 45-49 | 2090 | 150 | 180 | 201 | 225 | 268 | 205 | 173 |
| 50-54 | 2033 | 150 | 183 | 208 | 234 | 278 | 211 | 177 |
| 55-59 | 1701 | 154 | 187 | 212 | 239 | 287 | 215 | 180 |
| 60-64 | 2313 | 152 | 185 | 209 | 237 | 281 | 213 | 179 |
| 65-69 | 1703 | 140 | 181 | 206 | 233 | 281 | 209 | 174 |
| 70-74 | 1517 | 139 | 176 | 206 | 234 | 280 | 208 | 170 |
| 75-79 | 1057 | 141 | 176 | 201 | 228 | 275 | 204 | 169 |
| 80+ | 1778 | 136 | 173 | 199 | 229 | 275 | 202 | 166 |
| **HDL (mg/dL)** | | | | | | | | |
| 45-49 | 2090 | 35 | 46 | 56 | 67 | 90 | 58 | 44 |
| 50-54 | 2033 | 36 | 47 | 57 | 69 | 93 | 60 | 45 |
| 55-59 | 1701 | 37 | 47 | 58 | 70 | 93 | 61 | 46 |
| 60-64 | 2312 | 36 | 47 | 57 | 69 | 93 | 60 | 45 |
| 65-69 | 1703 | 35 | 48 | 58 | 70 | 90 | 60 | 45 |
| 70-74 | 1517 | 37 | 48 | 58 | 70 | 91 | 61 | 46 |
| 75-79 | 1057 | 36 | 47 | 57 | 70 | 90 | 60 | 45 |
| 80+ | 1777 | 37 | 48 | 58 | 71 | 92 | 61 | 46 |
| **LDL (mg/dL)** | | | | | | | | |
| 45-49 | 974 | 69 | 99 | 116 | 140 | 177 | 120 | 92 |
| 50-54 | 943 | 69 | 100 | 122 | 147 | 187 | 125 | 95 |
| 55-59 | 834 | 71 | 102 | 126 | 150 | 192 | 128 | 97 |
| 60-64 | 1123 | 69 | 98 | 120 | 144 | 187 | 123 | 94 |
| 65-69 | 813 | 59 | 92 | 115 | 142 | 183 | 120 | 86 |
| 70-74 | 716 | 56 | 89 | 115 | 144 | 187 | 119 | 85 |
| 75-79 | 497 | 59 | 90 | 110 | 136 | 170 | 114 | 84 |
| 80+ | 836 | 58 | 86 | 110 | 137 | 179 | 113 | 80 |
| **Triglycerides (mg/dL)** | | | | | | | | |
| 45-49 | 998 | 44 | 69 | 97 | 145 | 254 | 121 | 64 |
| 50-54 | 979 | 43 | 73 | 108 | 154 | 265 | 130 | 67 |
| 55-59 | 856 | 50 | 83 | 111 | 169 | 302 | 138 | 75 |
| 60-64 | 1141 | 51 | 81 | 113 | 168 | 272 | 135 | 74 |
| 65-69 | 847 | 51 | 79 | 116 | 167 | 274 | 137 | 74 |
| 70-74 | 745 | 57 | 88 | 124 | 170 | 265 | 138 | 81 |
| 75-79 | 521 | 55 | 86 | 116 | 164 | 272 | 136 | 78 |
| 80+ | 866 | 54 | 83 | 118 | 158 | 263 | 131 | 77 |

C. **NAHSIT**

|  | Observations | Percentiles | | | | | Mean | 20th |
| --- | --- | --- | --- | --- | --- | --- | --- | --- |
|  |  | **5th** | **25th** | **50th** | **75th** | **95th** |  |  |
| **CRP (mg/dL)** | | | | | | | | |
| 45-49 | 273 | 0.03 | 0.04 | 0.10 | 0.16 | 0.68 | 0.17 | 0.03 |
| 50-54 | 303 | 0.03 | 0.05 | 0.10 | 0.20 | 0.64 | 0.19 | 0.04 |
| 55-59 | 352 | 0.03 | 0.06 | 0.10 | 0.18 | 0.54 | 0.18 | 0.05 |
| 60-64 | 350 | 0.03 | 0.05 | 0.10 | 0.20 | 0.54 | 0.21 | 0.04 |
| 65-69 | 626 | 0.03 | 0.05 | 0.10 | 0.21 | 0.74 | 0.23 | 0.04 |
| 70-74 | 404 | 0.03 | 0.06 | 0.10 | 0.20 | 0.75 | 0.22 | 0.05 |
| 75-79 | 282 | 0.03 | 0.06 | 0.11 | 0.23 | 0.73 | 0.24 | 0.05 |
| 80+ | 207 | 0.03 | 0.07 | 0.12 | 0.29 | 0.87 | 0.32 | 0.06 |
| **Hb1AC (%)** | | | | | | | | |
| 45-49 | 272 | 4.9 | 5.3 | 5.5 | 5.8 | 6.5 | 5.6 | 5.2 |
| 50-54 | 303 | 5.1 | 5.4 | 5.6 | 5.9 | 6.8 | 5.8 | 5.4 |
| 55-59 | 352 | 5.1 | 5.5 | 5.8 | 6.1 | 7.9 | 6.0 | 5.4 |
| 60-64 | 348 | 5.2 | 5.5 | 5.8 | 6.2 | 7.7 | 6.0 | 5.5 |
| 65-69 | 627 | 5.1 | 5.6 | 5.8 | 6.3 | 7.9 | 6.1 | 5.5 |
| 70-74 | 404 | 5.2 | 5.6 | 5.9 | 6.5 | 8.1 | 6.2 | 5.5 |
| 75-79 | 285 | 5.2 | 5.6 | 5.9 | 6.5 | 8.2 | 6.2 | 5.5 |
| 80+ | 209 | 5.1 | 5.6 | 5.9 | 6.5 | 8.7 | 6.2 | 5.5 |
| **Glucose (mg/dL)** | | | | | | | | |
| 45-49 | 271 | 82 | 90 | 95 | 102 | 132 | 100 | 89 |
| 50-54 | 300 | 84 | 92 | 97 | 105 | 140 | 103 | 91 |
| 55-59 | 350 | 85 | 94 | 100 | 109 | 166 | 109 | 92 |
| 60-64 | 348 | 85 | 93 | 101 | 110 | 149 | 108 | 92 |
| 65-69 | 626 | 86 | 94 | 101 | 113 | 167 | 110 | 93 |
| 70-74 | 400 | 86 | 95 | 102 | 121 | 183 | 114 | 93 |
| 75-79 | 278 | 85 | 94 | 104 | 125 | 177 | 115 | 92 |
| 80+ | 206 | 86 | 94 | 103 | 124 | 218 | 118 | 93 |
| **Total cholesterol (mg/dL)** | | | | | | | | |
| 45-49 | 273 | 137 | 170 | 191 | 215 | 244 | 192 | 166 |
| 50-54 | 303 | 148 | 174 | 201 | 221 | 262 | 200 | 170 |
| 55-59 | 352 | 146 | 177 | 201 | 223 | 262 | 202 | 172 |
| 60-64 | 350 | 142 | 171 | 197 | 221 | 265 | 199 | 166 |
| 65-69 | 626 | 138 | 167 | 194 | 220 | 258 | 195 | 162 |
| 70-74 | 404 | 133 | 160 | 186 | 208 | 251 | 187 | 153 |
| 75-79 | 282 | 130 | 157 | 183 | 209 | 254 | 185 | 152 |
| 80+ | 207 | 127 | 155 | 178 | 205 | 249 | 181 | 150 |
| **HDL (mg/dL)** | | | | | | | | |
| 45-49 | 273 | 36 | 47 | 58 | 67 | 85 | 58 | 45 |
| 50-54 | 303 | 38 | 48 | 57 | 66 | 89 | 59 | 46 |
| 55-59 | 352 | 35 | 49 | 57 | 67 | 88 | 58 | 47 |
| 60-64 | 350 | 36 | 47 | 55 | 66 | 80 | 57 | 45 |
| 65-69 | 626 | 36 | 46 | 56 | 67 | 85 | 58 | 45 |
| 70-74 | 404 | 37 | 46 | 55 | 64 | 83 | 57 | 44 |
| 75-79 | 282 | 35 | 44 | 52 | 63 | 81 | 55 | 43 |
| 80+ | 207 | 32 | 43 | 54 | 63 | 83 | 54 | 41 |
| **LDL (mg/dL)** | | | | | | | | |
| 45-49 | 273 | 71 | 99 | 117 | 138 | 166 | 118 | 94 |
| 50-54 | 303 | 74 | 105 | 125 | 147 | 181 | 126 | 97 |
| 55-59 | 352 | 74 | 100 | 126 | 147 | 181 | 126 | 96 |
| 60-64 | 350 | 71 | 98 | 126 | 148 | 188 | 125 | 93 |
| 65-69 | 626 | 69 | 95 | 120 | 148 | 186 | 123 | 91 |
| 70-74 | 404 | 66 | 91 | 112 | 136 | 169 | 116 | 87 |
| 75-79 | 282 | 65 | 89 | 113 | 136 | 171 | 115 | 85 |
| 80+ | 207 | 59 | 82 | 109 | 132 | 169 | 109 | 78 |
| **Triglycerides (mg/dL)** | | | | | | | | |
| 45-49 | 273 | 45 | 67 | 95 | 139 | 301 | 124 | 61 |
| 50-54 | 303 | 47 | 75 | 104 | 150 | 261 | 124 | 69 |
| 55-59 | 352 | 51 | 81 | 106 | 154 | 276 | 137 | 77 |
| 60-64 | 350 | 57 | 82 | 118 | 154 | 255 | 131 | 77 |
| 65-69 | 626 | 54 | 80 | 108 | 149 | 267 | 128 | 73 |
| 70-74 | 404 | 52 | 82 | 108 | 146 | 236 | 122 | 76 |
| 75-79 | 282 | 58 | 83 | 113 | 152 | 259 | 129 | 76 |
| 80+ | 207 | 63 | 87 | 113 | 155 | 270 | 134 | 84 |

CRP, C-reactive protein; Hb1AC, hemoglobin A1C; HDL, high-density lipoprotein; LDL, low-density lipoprotein

For NHANES, all analyses were performed using the combined sample weights in accordance with the analytical guidelines.

**Table 6. Age-specific normative values for muscle-related biomarkers among men**

A. **UK biobank**

|  | Observations | Percentiles | | | | | Mean | 20th |
| --- | --- | --- | --- | --- | --- | --- | --- | --- |
|  |  | **5th** | **25th** | **50th** | **75th** | **95th** |  |  |
| **CRP (mg/dL)** | | | | | | | | |
| 45-49 | 720 | 0.02 | 0.05 | 0.10 | 0.20 | 0.62 | 0.20 | 0.04 |
| 50-54 | 950 | 0.03 | 0.05 | 0.10 | 0.19 | 0.59 | 0.19 | 0.05 |
| 55-59 | 1200 | 0.03 | 0.06 | 0.11 | 0.22 | 0.63 | 0.21 | 0.05 |
| 60-64 | 2059 | 0.03 | 0.06 | 0.12 | 0.23 | 0.70 | 0.22 | 0.06 |
| 65-69 | 2560 | 0.03 | 0.07 | 0.13 | 0.24 | 0.78 | 0.24 | 0.06 |
| 70-74 | 1396 | 0.03 | 0.07 | 0.13 | 0.26 | 0.92 | 0.28 | 0.06 |
| 75-79 | 39 | 0.02 | 0.06 | 0.09 | 0.36 | 2.61 | 0.38 | 0.05 |
| 80+ | NA | NA | NA | NA | NA | NA | NA | NA |
| **Hb1AC (%)** | | | | | | | | |
| 45-49 | 555 | 4.8 | 5.1 | 5.3 | 5.5 | 5.9 | 5.3 | 5.0 |
| 50-54 | 701 | 4.8 | 5.1 | 5.3 | 5.5 | 6.1 | 5.4 | 5.1 |
| 55-59 | 913 | 4.9 | 5.2 | 5.4 | 5.6 | 6.3 | 5.5 | 5.1 |
| 60-64 | 1602 | 4.9 | 5.2 | 5.4 | 5.6 | 6.4 | 5.5 | 5.1 |
| 65-69 | 2042 | 4.9 | 5.2 | 5.5 | 5.7 | 6.5 | 5.5 | 5.2 |
| 70-74 | 1087 | 5.0 | 5.3 | 5.5 | 5.8 | 6.7 | 5.6 | 5.3 |
| 75-79 | 35 | 4.9 | 5.2 | 5.5 | 5.9 | 6.9 | 5.6 | 5.2 |
| 80+ | NA | NA | NA | NA | NA | NA | NA | NA |
| **Glucose (mg/dL)** | | | | | | | | |
| 45-49 | 646 | 72 | 81 | 87 | 92 | 106 | 88 | 80 |
| 50-54 | 863 | 73 | 82 | 88 | 94 | 109 | 90 | 81 |
| 55-59 | 1066 | 73 | 83 | 89 | 95 | 113 | 92 | 81 |
| 60-64 | 1820 | 74 | 84 | 89 | 96 | 115 | 92 | 82 |
| 65-69 | 2265 | 74 | 84 | 90 | 97 | 120 | 93 | 82 |
| 70-74 | 1231 | 75 | 84 | 91 | 98 | 126 | 95 | 82 |
| 75-79 | 35 | 77 | 89 | 95 | 102 | 126 | 97 | 88 |
| 80+ | NA | NA | NA | NA | NA | NA | NA | NA |
| **Total cholesterol (mg/dL)** | | | | | | | | |
| 45-49 | 720 | 159 | 192 | 217 | 247 | 292 | 220 | 184 |
| 50-54 | 954 | 151 | 189 | 216 | 249 | 292 | 218 | 184 |
| 55-59 | 1203 | 142 | 186 | 214 | 246 | 289 | 216 | 179 |
| 60-64 | 2066 | 138 | 181 | 212 | 244 | 282 | 212 | 173 |
| 65-69 | 2562 | 133 | 169 | 199 | 230 | 274 | 201 | 162 |
| 70-74 | 1400 | 133 | 165 | 192 | 226 | 270 | 196 | 158 |
| 75-79 | 39 | 121 | 151 | 164 | 204 | 252 | 175 | 142 |
| 80+ | NA | NA | NA | NA | NA | NA | NA | NA |
| **HDL (mg/dL)** | | | | | | | | |
| 45-49 | 645 | 34 | 42 | 49 | 58 | 72 | 51 | 41 |
| 50-54 | 863 | 34 | 42 | 50 | 58 | 73 | 51 | 40 |
| 55-59 | 1066 | 35 | 44 | 51 | 60 | 75 | 53 | 42 |
| 60-64 | 1822 | 35 | 44 | 51 | 60 | 78 | 53 | 42 |
| 65-69 | 2265 | 35 | 43 | 50 | 59 | 76 | 52 | 42 |
| 70-74 | 1231 | 35 | 43 | 51 | 60 | 76 | 53 | 42 |
| 75-79 | 35 | 37 | 44 | 54 | 61 | 84 | 55 | 43 |
| 80+ | NA | NA | NA | NA | NA | NA | NA | NA |
| **LDL (mg/dL)** | | | | | | | | |
| 45-49 | 719 | 95 | 118 | 140 | 161 | 194 | 141 | 113 |
| 50-54 | 953 | 90 | 118 | 137 | 161 | 195 | 140 | 113 |
| 55-59 | 1201 | 84 | 113 | 137 | 161 | 192 | 138 | 108 |
| 60-64 | 2062 | 78 | 109 | 134 | 158 | 188 | 134 | 103 |
| 65-69 | 2559 | 74 | 100 | 123 | 148 | 182 | 125 | 95 |
| 70-74 | 1393 | 74 | 97 | 118 | 144 | 177 | 121 | 92 |
| 75-79 | 39 | 66 | 86 | 96 | 117 | 157 | 103 | 86 |
| 80+ | NA | NA | NA | NA | NA | NA | NA | NA |
| **Triglycerides (mg/dL)** | | | | | | | | |
| 45-49 | 717 | 62 | 98 | 148 | 227 | 396 | 180 | 91 |
| 50-54 | 954 | 65 | 104 | 147 | 218 | 397 | 178 | 94 |
| 55-59 | 1203 | 65 | 103 | 144 | 205 | 352 | 167 | 95 |
| 60-64 | 2060 | 69 | 104 | 144 | 206 | 334 | 166 | 96 |
| 65-69 | 2557 | 67 | 99 | 137 | 191 | 305 | 156 | 93 |
| 70-74 | 1396 | 64 | 95 | 130 | 179 | 292 | 149 | 87 |
| 75-79 | 39 | 45 | 79 | 107 | 144 | 257 | 124 | 72 |
| 80+ | NA | NA | NA | NA | NA | NA | NA | NA |

B. **NHANES**

|  | Observations | Percentiles | | | | | Mean | 20th |
| --- | --- | --- | --- | --- | --- | --- | --- | --- |
|  |  | **5th** | **25th** | **50th** | **75th** | **95th** |  |  |
| **CRP (mg/dL)** | | | | | | | | |
| 45-49 | 1161 | 0.02 | 0.07 | 0.16 | 0.32 | 1.28 | 0.40 | 0.06 |
| 50-54 | 1190 | 0.02 | 0.07 | 0.17 | 0.35 | 1.06 | 0.33 | 0.06 |
| 55-59 | 847 | 0.02 | 0.07 | 0.18 | 0.40 | 1.22 | 0.35 | 0.06 |
| 60-64 | 1217 | 0.03 | 0.08 | 0.19 | 0.41 | 1.29 | 0.40 | 0.07 |
| 65-69 | 1010 | 0.03 | 0.08 | 0.19 | 0.43 | 1.55 | 0.46 | 0.07 |
| 70-74 | 950 | 0.03 | 0.09 | 0.21 | 0.42 | 1.67 | 0.52 | 0.08 |
| 75-79 | 729 | 0.03 | 0.09 | 0.21 | 0.47 | 1.50 | 0.47 | 0.07 |
| 80+ | 1013 | 0.03 | 0.10 | 0.23 | 0.50 | 1.86 | 0.52 | 0.08 |
| **Hb1AC (%)** | | | | | | | | |
| 45-49 | 1951 | 4.8 | 5.2 | 5.4 | 5.7 | 7.5 | 5.7 | 5.1 |
| 50-54 | 2031 | 4.9 | 5.2 | 5.4 | 5.7 | 7.9 | 5.8 | 5.1 |
| 55-59 | 1658 | 4.9 | 5.3 | 5.5 | 5.9 | 8.0 | 5.9 | 5.2 |
| 60-64 | 2273 | 4.9 | 5.3 | 5.6 | 6.0 | 8.2 | 6.0 | 5.2 |
| 65-69 | 1771 | 5.0 | 5.4 | 5.7 | 6.2 | 8.3 | 6.0 | 5.3 |
| 70-74 | 1566 | 5.0 | 5.4 | 5.7 | 6.1 | 7.9 | 6.0 | 5.3 |
| 75-79 | 1153 | 5.0 | 5.4 | 5.7 | 6.2 | 7.7 | 6.0 | 5.3 |
| 80+ | 1647 | 5.0 | 5.4 | 5.7 | 6.1 | 7.5 | 5.9 | 5.3 |
| **Glucose (mg/dL)** | | | | | | | | |
| 45-49 | 979 | 87 | 95 | 101 | 110 | 151 | 108 | 94 |
| 50-54 | 981 | 88 | 97 | 104 | 114 | 198 | 116 | 96 |
| 55-59 | 822 | 88 | 97 | 104 | 113 | 162 | 112 | 95 |
| 60-64 | 1121 | 87 | 98 | 106 | 119 | 203 | 118 | 97 |
| 65-69 | 861 | 89 | 99 | 109 | 123 | 200 | 119 | 96 |
| 70-74 | 786 | 88 | 99 | 109 | 126 | 197 | 120 | 97 |
| 75-79 | 534 | 89 | 100 | 109 | 124 | 189 | 120 | 97 |
| 80+ | 797 | 88 | 99 | 107 | 121 | 172 | 115 | 97 |
| **Total cholesterol (mg/dL)** | | | | | | | | |
| 45-49 | 1943 | 144 | 178 | 202 | 230 | 278 | 207 | 173 |
| 50-54 | 2009 | 139 | 172 | 201 | 229 | 274 | 203 | 166 |
| 55-59 | 1638 | 134 | 173 | 199 | 225 | 271 | 201 | 166 |
| 60-64 | 2245 | 130 | 164 | 190 | 218 | 268 | 193 | 156 |
| 65-69 | 1749 | 123 | 157 | 185 | 213 | 255 | 187 | 152 |
| 70-74 | 1551 | 121 | 153 | 178 | 206 | 252 | 181 | 146 |
| 75-79 | 1138 | 122 | 152 | 177 | 206 | 250 | 181 | 145 |
| 80+ | 1620 | 118 | 146 | 173 | 199 | 240 | 175 | 141 |
| **HDL (mg/dL)** | | | | | | | | |
| 45-49 | 1943 | 30 | 37 | 44 | 53 | 71 | 47 | 36 |
| 50-54 | 2009 | 29 | 37 | 45 | 54 | 71 | 47 | 36 |
| 55-59 | 1638 | 30 | 38 | 45 | 56 | 75 | 49 | 36 |
| 60-64 | 2245 | 29 | 38 | 45 | 54 | 75 | 48 | 36 |
| 65-69 | 1748 | 29 | 39 | 45 | 57 | 78 | 49 | 37 |
| 70-74 | 1551 | 30 | 39 | 45 | 55 | 74 | 49 | 37 |
| 75-79 | 1138 | 30 | 39 | 47 | 56 | 75 | 49 | 37 |
| 80+ | 1620 | 31 | 40 | 47 | 57 | 78 | 50 | 38 |
| **LDL (mg/dL)** | | | | | | | | |
| 45-49 | 914 | 70 | 104 | 124 | 148 | 186 | 127 | 99 |
| 50-54 | 903 | 70 | 100 | 123 | 146 | 183 | 125 | 96 |
| 55-59 | 784 | 66 | 96 | 117 | 139 | 180 | 119 | 91 |
| 60-64 | 1037 | 57 | 89 | 112 | 134 | 177 | 114 | 83 |
| 65-69 | 814 | 55 | 81 | 106 | 132 | 172 | 109 | 78 |
| 70-74 | 756 | 54 | 78 | 102 | 126 | 167 | 105 | 73 |
| 75-79 | 501 | 51 | 79 | 99 | 126 | 166 | 104 | 74 |
| 80+ | 757 | 52 | 75 | 96 | 122 | 158 | 100 | 71 |
| **Triglycerides (mg/dL)** | | | | | | | | |
| 45-49 | 967 | 53 | 88 | 125 | 185 | 381 | 160 | 79 |
| 50-54 | 960 | 48 | 83 | 123 | 188 | 384 | 166 | 75 |
| 55-59 | 813 | 50 | 82 | 122 | 181 | 323 | 154 | 74 |
| 60-64 | 1099 | 49 | 78 | 113 | 166 | 331 | 144 | 71 |
| 65-69 | 849 | 51 | 85 | 116 | 170 | 271 | 138 | 77 |
| 70-74 | 776 | 50 | 84 | 115 | 165 | 279 | 135 | 77 |
| 75-79 | 518 | 52 | 78 | 109 | 153 | 262 | 130 | 69 |
| 80+ | 779 | 48 | 75 | 101 | 146 | 243 | 121 | 69 |

C. **NAHSIT**

|  | Observations | Percentiles | | | | | Mean | 20th |
| --- | --- | --- | --- | --- | --- | --- | --- | --- |
|  |  | **5th** | **25th** | **50th** | **75th** | **95th** |  |  |
| **CRP (mg/dL)** | | | | | | | | |
| 45-49 | 204 | 0.03 | 0.05 | 0.10 | 0.19 | 0.67 | 0.18 | 0.05 |
| 50-54 | 267 | 0.03 | 0.05 | 0.10 | 0.20 | 0.51 | 0.16 | 0.04 |
| 55-59 | 357 | 0.03 | 0.05 | 0.10 | 0.19 | 0.73 | 0.21 | 0.04 |
| 60-64 | 368 | 0.03 | 0.05 | 0.10 | 0.19 | 0.71 | 0.22 | 0.04 |
| 65-69 | 628 | 0.03 | 0.05 | 0.10 | 0.19 | 0.58 | 0.22 | 0.04 |
| 70-74 | 406 | 0.03 | 0.05 | 0.10 | 0.19 | 0.79 | 0.21 | 0.04 |
| 75-79 | 300 | 0.03 | 0.05 | 0.10 | 0.21 | 1.01 | 0.27 | 0.05 |
| 80+ | 283 | 0.03 | 0.08 | 0.12 | 0.25 | 0.95 | 0.28 | 0.06 |
| **Hb1AC (%)** | | | | | | | | |
| 45-49 | 204 | 4.7 | 5.3 | 5.6 | 5.9 | 6.8 | 5.7 | 5.3 |
| 50-54 | 267 | 5.0 | 5.4 | 5.7 | 6.1 | 7.8 | 5.9 | 5.4 |
| 55-59 | 356 | 5.1 | 5.5 | 5.8 | 6.1 | 8.0 | 6.0 | 5.4 |
| 60-64 | 368 | 5.2 | 5.5 | 5.8 | 6.3 | 8.5 | 6.1 | 5.5 |
| 65-69 | 627 | 5.1 | 5.5 | 5.9 | 6.4 | 8.4 | 6.1 | 5.5 |
| 70-74 | 408 | 5.2 | 5.6 | 5.9 | 6.4 | 7.5 | 6.1 | 5.5 |
| 75-79 | 298 | 5.0 | 5.6 | 5.9 | 6.4 | 8.0 | 6.2 | 5.5 |
| 80+ | 280 | 5.1 | 5.6 | 5.9 | 6.2 | 7.7 | 6.0 | 5.5 |
| **Glucose (mg/dL)** | | | | | | | | |
| 45-49 | 204 | 86 | 93.5 | 100 | 107 | 137 | 105 | 92 |
| 50-54 | 266 | 86 | 94 | 101 | 111 | 165 | 109 | 92 |
| 55-59 | 357 | 86 | 94 | 101 | 112 | 171 | 111 | 93 |
| 60-64 | 366 | 86 | 96 | 104 | 119 | 170 | 114 | 94 |
| 65-69 | 626 | 87 | 96 | 105 | 118 | 165 | 113 | 94 |
| 70-74 | 406 | 85 | 96 | 105 | 120 | 173 | 115 | 94 |
| 75-79 | 297 | 86 | 95 | 103 | 121 | 178 | 113 | 93 |
| 80+ | 279 | 89 | 96 | 102 | 115 | 166 | 112 | 95 |
| **Total cholesterol (mg/dL)** | | | | | | | | |
| 45-49 | 204 | 140 | 166 | 192 | 216 | 257 | 194 | 161 |
| 50-54 | 267 | 134 | 169 | 187 | 206 | 242 | 188 | 164 |
| 55-59 | 357 | 129 | 163 | 186 | 212 | 254 | 188 | 158 |
| 60-64 | 368 | 129 | 162 | 185 | 210 | 261 | 187 | 156 |
| 65-69 | 628 | 124 | 151 | 174 | 198 | 240 | 176 | 145 |
| 70-74 | 406 | 120 | 147 | 171 | 196 | 239 | 173 | 142 |
| 75-79 | 300 | 117 | 146 | 168 | 190 | 226 | 169 | 142 |
| 80+ | 283 | 121 | 146 | 167 | 193 | 225 | 170 | 143 |
| **HDL (mg/dL)** | | | | | | | | |
| 45-49 | 204 | 31 | 38 | 46 | 51 | 69 | 46 | 37 |
| 50-54 | 267 | 30 | 38 | 46 | 54 | 73 | 47 | 37 |
| 55-59 | 357 | 30 | 38 | 45 | 53 | 72 | 47 | 36 |
| 60-64 | 368 | 30 | 39 | 46 | 56 | 76 | 48 | 37 |
| 65-69 | 628 | 30 | 38 | 45 | 54 | 69 | 47 | 37 |
| 70-74 | 406 | 31 | 38 | 46 | 56 | 72 | 48 | 37 |
| 75-79 | 300 | 28 | 38 | 47 | 56 | 72 | 48 | 37 |
| 80+ | 283 | 30 | 40 | 48 | 57 | 72 | 49 | 38 |
| **LDL (mg/dL)** | | | | | | | | |
| 45-49 | 204 | 68 | 102 | 121 | 148 | 176 | 123 | 98 |
| 50-54 | 267 | 68 | 99 | 118 | 137 | 173 | 119 | 94 |
| 55-59 | 357 | 69 | 98 | 119 | 143 | 178 | 120 | 91 |
| 60-64 | 368 | 65 | 95 | 119 | 141 | 182 | 119 | 92 |
| 65-69 | 628 | 62 | 90 | 112 | 135 | 167 | 113 | 85 |
| 70-74 | 406 | 59 | 87 | 108 | 132 | 164 | 110 | 81 |
| 75-79 | 300 | 54 | 82 | 105 | 128 | 159 | 106 | 78 |
| 80+ | 283 | 60 | 84 | 105 | 127 | 156 | 107 | 80 |
| **Triglycerides (mg/dL)** | | | | | | | | |
| 45-49 | 204 | 59 | 91 | 126 | 194 | 348 | 171 | 83 |
| 50-54 | 267 | 58 | 89 | 122 | 185 | 338 | 154 | 84 |
| 55-59 | 357 | 55 | 90 | 125 | 173 | 341 | 151 | 80 |
| 60-64 | 368 | 54 | 85 | 116 | 171 | 310 | 145 | 78 |
| 65-69 | 628 | 56 | 84 | 115 | 164 | 280 | 134 | 78 |
| 70-74 | 406 | 53 | 78 | 106 | 141 | 258 | 124 | 70 |
| 75-79 | 300 | 50 | 76 | 106 | 145 | 294 | 125 | 71 |
| 80+ | 283 | 49 | 71 | 94 | 138 | 210 | 113 | 67 |

CRP, C-reactive protein; Hb1AC, hemoglobin A1C; HDL, high-density lipoprotein; LDL, low-density lipoprotein

For NHANES, all analyses were performed using the combined sample weights in accordance with the analytical guidelines.

**Table 7. Age-specific normative values for lipid metabolism-related biomarkers among women**

**UK biobank**

|  | Observations | Percentiles | | | | | Mean | 20th |
| --- | --- | --- | --- | --- | --- | --- | --- | --- |
|  |  | **5th** | **25th** | **50th** | **75th** | **95th** |  |  |
| **β-Hydroxybutyrate (mmol/L)** | | | | | | | | |
| 45-49 | 743 | 0.006 | 0.018 | 0.032 | 0.059 | 0.145 | 0.048 | 0.015 |
| 50-54 | 991 | 0.008 | 0.022 | 0.036 | 0.060 | 0.141 | 0.051 | 0.019 |
| 55-59 | 1465 | 0.010 | 0.025 | 0.039 | 0.065 | 0.161 | 0.055 | 0.022 |
| 60-64 | 2051 | 0.011 | 0.026 | 0.039 | 0.065 | 0.160 | 0.055 | 0.023 |
| 65-69 | 2131 | 0.011 | 0.029 | 0.042 | 0.069 | 0.164 | 0.058 | 0.023 |
| 70-74 | 883 | 0.012 | 0.027 | 0.042 | 0.073 | 0.175 | 0.061 | 0.023 |
| 75-79 | 24 | 0.016 | 0.028 | 0.043 | 0.073 | 0.124 | 0.053 | 0.026 |
| 80+ | NA | NA | NA | NA | NA | NA | NA | NA |
| **Acetoacetate (mmol/L)** | | | | | | | | |
| 45-49 | 758 | 0.002 | 0.007 | 0.011 | 0.019 | 0.036 | 0.014 | 0.006 |
| 50-54 | 999 | 0.003 | 0.007 | 0.012 | 0.018 | 0.040 | 0.015 | 0.006 |
| 55-59 | 1483 | 0.003 | 0.008 | 0.013 | 0.020 | 0.040 | 0.017 | 0.007 |
| 60-64 | 2099 | 0.003 | 0.008 | 0.013 | 0.020 | 0.041 | 0.017 | 0.008 |
| 65-69 | 2169 | 0.004 | 0.009 | 0.014 | 0.021 | 0.043 | 0.017 | 0.008 |
| 70-74 | 905 | 0.004 | 0.009 | 0.015 | 0.023 | 0.049 | 0.019 | 0.008 |
| 75-79 | 24 | 0.007 | 0.011 | 0.015 | 0.024 | 0.039 | 0.018 | 0.009 |
| 80+ | NA | NA | NA | NA | NA | NA | NA | NA |
| **Acetone (mmol/L)** | | | | | | | | |
| 45-49 | 758 | 0.009 | 0.010 | 0.012 | 0.014 | 0.021 | 0.013 | 0.010 |
| 50-54 | 999 | 0.009 | 0.011 | 0.012 | 0.014 | 0.020 | 0.013 | 0.010 |
| 55-59 | 1483 | 0.009 | 0.011 | 0.012 | 0.015 | 0.021 | 0.013 | 0.010 |
| 60-64 | 2099 | 0.009 | 0.011 | 0.012 | 0.015 | 0.022 | 0.013 | 0.010 |
| 65-69 | 2169 | 0.009 | 0.011 | 0.012 | 0.015 | 0.022 | 0.013 | 0.010 |
| 70-74 | 905 | 0.009 | 0.011 | 0.013 | 0.015 | 0.021 | 0.014 | 0.011 |
| 75-79 | 24 | 0.009 | 0.011 | 0.012 | 0.015 | 0.023 | 0.013 | 0.010 |
| 80+ | NA | NA | NA | NA | NA | NA | NA | NA |
| **IGF-1 (mmol/L)** | | | | | | | | |
| 45-49 | 761 | 14.22 | 19.34 | 22.89 | 26.48 | 34.03 | 23.16 | 18.30 |
| 50-54 | 1025 | 12.89 | 17.75 | 21.53 | 24.94 | 31.94 | 21.72 | 16.88 |
| 55-59 | 1550 | 12.24 | 16.93 | 20.46 | 24.12 | 29.97 | 20.66 | 15.93 |
| 60-64 | 2206 | 11.93 | 16.32 | 19.89 | 23.24 | 29.21 | 20.07 | 15.57 |
| 65-69 | 2228 | 11.46 | 15.71 | 18.99 | 22.74 | 28.27 | 19.38 | 14.89 |
| 70-74 | 980 | 11.53 | 15.03 | 18.20 | 21.98 | 27.32 | 18.75 | 14.26 |
| 75-79 | 27 | 10.10 | 13.41 | 17.50 | 22.21 | 27.03 | 17.64 | 12.90 |
| 80+ | NA | NA | NA | NA | NA | NA | NA | NA |
| **IL-6 (NPX)** | | | | | | | | |
| 45-49 | 39 | -1.25 | -0.73 | -0.11 | 0.31 | 1.32 | -0.03 | -0.79 |
| 50-54 | 149 | -1.17 | -0.63 | -0.23 | 0.20 | 1.4 | -0.14 | -0.74 |
| 55-59 | 175 | -0.93 | -0.38 | -0.01 | 0.41 | 1.35 | 0.07 | -0.47 |
| 60-64 | 126 | -0.94 | 0.42 | 0.03 | 0.48 | 1.24 | 0.07 | -0.54 |
| 65-69 | 66 | -1.09 | -0.22 | 0.15 | 0.59 | 1.73 | 0.23 | -0.35 |
| 70-74 | 42 | -0.43 | -0.12 | 0.26 | 0.72 | 1.72 | 0.40 | -0.18 |
| 75-79 | 12 | -0.86 | 0.07 | 0.45 | 0.92 | 2.08 | 0.50 | -0.04 |
| 80+ | NA | NA | NA | NA | NA | NA | NA | NA |
| **IL-1beta (NPX)** | | | | | | | | |
| 45-49 | 38 | -1.50 | -0.86 | -0.41 | -0.01 | 1.23 | -0.33 | -0.93 |
| 50-54 | 150 | -1.14 | -0.56 | -0.23 | 0.13 | 0.81 | -0.19 | -0.62 |
| 55-59 | 177 | -1.02 | -0.47 | -0.13 | 0.34 | 1.06 | -0.06 | -0.57 |
| 60-64 | 124 | -0.94 | -0.42 | -0.04 | 0.32 | 1.03 | -0.01 | -0.53 |
| 65-69 | 66 | -0.97 | -0.41 | 0.00 | 0.24 | 1.16 | -0.02 | -0.48 |
| 70-74 | 42 | -1.06 | -0.52 | -0.01 | 0.21 | 0.76 | -0.13 | -0.71 |
| 75-79 | 12 | -0.94 | -0.49 | -0.20 | 0.16 | 0.52 | -0.23 | -0.54 |
| 80+ | NA | NA | NA | NA | NA | NA | NA | NA |
| **TNF (NPX)** | | | | | | | | |
| 45-49 | 36 | -0.70 | -0.44 | -0.23 | 0.02 | 0.24 | -0.22 | -0.46 |
| 50-54 | 151 | -0.63 | -0.34 | -0.13 | 0.07 | 0.59 | -0.11 | -0.38 |
| 55-59 | 178 | -0.45 | -0.22 | -0.02 | 0.26 | 0.92 | 0.08 | -0.28 |
| 60-64 | 125 | -0.49 | -0.22 | -0.03 | 0.20 | 0.63 | 0.01 | -0.24 |
| 65-69 | 67 | -0.46 | -0.10 | 0.00 | 0.19 | 0.71 | 0.05 | -0.12 |
| 70-74 | 41 | -0.39 | -0.13 | 0.20 | 0.45 | 0.61 | 0.16 | -0.21 |
| 75-79 | 12 | -0.33 | 0.05 | 0.20 | 0.42 | 0.68 | 0.22 | 0.04 |
| 80+ | NA | NA | NA | NA | NA | NA | NA | NA |

IGF-1, insulin-like growth factor-1; IL-6, interleukin-6; IL-1beta, interleukin-1β; TNF, tumor necrosis factor; NPX, normalized protein expression

**Table 8. Age-specific normative values for lipid metabolism-related biomarkers among men**

**UK biobank**

|  | Observations | Percentiles | | | | | Mean | 20th |
| --- | --- | --- | --- | --- | --- | --- | --- | --- |
|  |  | **5th** | **25th** | **50th** | **75th** | **95th** |  |  |
| **β-Hydroxybutyrate (mmol/L)** | | | | | | | | |
| 45-49 | 665 | 0.011 | 0.025 | 0.038 | 0.054 | 0.127 | 0.049 | 0.022 |
| 50-54 | 837 | 0.012 | 0.025 | 0.037 | 0.051 | 0.127 | 0.050 | 0.023 |
| 55-59 | 1090 | 0.014 | 0.027 | 0.039 | 0.059 | 0.146 | 0.054 | 0.024 |
| 60-64 | 1861 | 0.015 | 0.029 | 0.041 | 0.063 | 0.155 | 0.057 | 0.026 |
| 65-69 | 2318 | 0.015 | 0.029 | 0.042 | 0.067 | 0.173 | 0.062 | 0.026 |
| 70-74 | 1245 | 0.015 | 0.030 | 0.045 | 0.070 | 0.175 | 0.063 | 0.028 |
| 75-79 | 31 | 0.015 | 0.036 | 0.051 | 0.082 | 0.450 | 0.091 | 0.035 |
| 80+ | NA | NA | NA | NA | NA | NA | NA | NA |
| **Acetoacetate (mmol/L)** | | | | | | | | |
| 45-49 | 681 | 0.003 | 0.007 | 0.013 | 0.019 | 0.037 | 0.015 | 0.007 |
| 50-54 | 857 | 0.004 | 0.008 | 0.013 | 0.020 | 0.038 | 0.016 | 0.007 |
| 55-59 | 1115 | 0.003 | 0.008 | 0.013 | 0.021 | 0.044 | 0.017 | 0.007 |
| 60-64 | 1902 | 0.003 | 0.008 | 0.014 | 0.021 | 0.043 | 0.017 | 0.007 |
| 65-69 | 2368 | 0.004 | 0.009 | 0.014 | 0.022 | 0.048 | 0.018 | 0.008 |
| 70-74 | 1258 | 0.003 | 0.009 | 0.014 | 0.023 | 0.048 | 0.019 | 0.008 |
| 75-79 | 31 | 0.005 | 0.010 | 0.016 | 0.028 | 0.112 | 0.027 | 0.009 |
| 80+ | NA | NA | NA | NA | NA | NA | NA | NA |
| **Acetone (mmol/L)** | | | | | | | | |
| 45-49 | 681 | 0.009 | 0.011 | 0.012 | 0.014 | 0.021 | 0.013 | 0.010 |
| 50-54 | 857 | 0.009 | 0.011 | 0.012 | 0.014 | 0.020 | 0.014 | 0.010 |
| 55-59 | 1115 | 0.009 | 0.011 | 0.013 | 0.015 | 0.023 | 0.014 | 0.011 |
| 60-64 | 1902 | 0.009 | 0.011 | 0.013 | 0.015 | 0.022 | 0.014 | 0.011 |
| 65-69 | 2368 | 0.009 | 0.011 | 0.013 | 0.015 | 0.023 | 0.014 | 0.011 |
| 70-74 | 1258 | 0.009 | 0.011 | 0.013 | 0.016 | 0.023 | 0.014 | 0.011 |
| 75-79 | 31 | 0.009 | 0.012 | 0.014 | 0.017 | 0.031 | 0.017 | 0.012 |
| 80+ | NA | NA | NA | NA | NA | NA | NA | NA |
| **IGF-1 (mmol/L)** | | | | | | | | |
| 45-49 | 714 | 15.59 | 20.50 | 23.77 | 27.14 | 33.58 | 23.97 | 19.66 |
| 50-54 | 947 | 14.75 | 19.04 | 22.46 | 25.88 | 31.81 | 22.69 | 18.22 |
| 55-59 | 1190 | 13.93 | 18.74 | 22.18 | 25.47 | 31.47 | 22.34 | 17.95 |
| 60-64 | 2048 | 13.27 | 18.12 | 21.39 | 24.79 | 29.97 | 21.57 | 17.29 |
| 65-69 | 2531 | 13.16 | 17.29 | 20.84 | 23.99 | 29.60 | 20.92 | 16.39 |
| 70-74 | 1385 | 11.94 | 16.42 | 20.12 | 23.48 | 29.28 | 20.20 | 15.47 |
| 75-79 | 39 | 9.37 | 13.63 | 19.05 | 22.99 | 31.64 | 19.11 | 13.14 |
| 80+ | NA | NA | NA | NA | NA | NA | NA | NA |
| **IL-6 (NPX)** | | | | | | | | |
| 45-49 | 24 | -0.69 | -0.31 | -0.10 | 0.76 | 0.99 | 0.15 | -0.46 |
| 50-54 | 124 | -1.05 | -0.64 | -0.20 | 0.52 | 1.57 | 0.02 | -0.75 |
| 55-59 | 141 | -1.02 | -0.52 | -0.08 | 0.32 | 1.00 | -0.04 | -0.59 |
| 60-64 | 80 | -0.60 | -0.24 | 0.02 | 0.57 | 1.66 | 0.25 | -0.31 |
| 65-69 | 76 | -0.68 | -0.11 | 0.37 | 0.99 | 2.51 | 0.50 | -0.14 |
| 70-74 | 54 | -0.69 | 0.02 | 0.37 | 0.96 | 2.58 | 0.56 | -0.23 |
| 75-79 | 31 | -0.55 | 0.08 | 0.38 | 1.46 | 2.66 | 0.63 | -0.11 |
| 80+ | NA | NA | NA | NA | NA | NA | NA | NA |
| **IL-1β (NPX)** | | | | | | | | |
| 45-49 | 23 | -1.82 | -1.09 | -0.81 | -0.44 | 1.41 | -0.69 | -1.17 |
| 50-54 | 118 | -1.14 | -0.63 | -0.29 | 0.11 | 0.77 | -0.26 | -0.73 |
| 55-59 | 138 | -1.10 | -0.51 | -0.17 | 0.20 | 0.87 | -0.12 | -0.63 |
| 60-64 | 80 | -1.04 | -0.60 | -0.26 | 0.15 | 1.02 | -0.18 | -0.63 |
| 65-69 | 75 | -1.10 | -0.56 | -0.10 | 0.25 | 0.76 | -0.14 | -0.62 |
| 70-74 | 52 | -1.11 | -0.48 | -0.15 | 0.17 | 0.52 | -0.18 | 0.56 |
| 75-79 | 30 | -1.15 | -0.46 | -0.21 | 0.10 | 0.51 | -0.18 | -0.47 |
| 80+ | NA | NA | NA | NA | NA | NA | NA | NA |
| **TNF (mg/dL)** | | | | | | | | |
| 45-49 | 24 | -0.51 | -0.28 | -0.07 | 0.20 | 0.66 | -0.01 | -0.33 |
| 50-54 | 122 | -0.75 | -0.32 | -0.13 | 0.11 | 0.65 | -0.06 | -0.38 |
| 55-59 | 140 | -0.49 | -0.28 | -0.07 | 0.16 | 0.49 | -0.04 | -0.30 |
| 60-64 | 78 | -0.61 | -0.19 | -0.01 | 0.12 | 0.55 | -0.02 | -0.21 |
| 65-69 | 78 | -0.51 | -0.21 | -0.03 | 0.23 | 0.64 | 0.01 | -0.27 |
| 70-74 | 51 | -0.42 | -0.21 | 0.03 | 0.19 | 0.62 | 0.05 | -0.23 |
| 75-79 | 31 | -0.38 | -0.14 | 0.06 | 0.28 | 0.74 | 0.10 | -0.18 |
| 80+ | NA | NA | NA | NA | NA | NA | NA | NA |

IGF-1, insulin-like growth factor-1; IL-6, interleukin-6; IL-1beta, interleukin-1β; TNF, tumor necrosis factor; NPX, normalized protein expression

**Supplementary Figure 1. Age- specific normative values for lipid profiles**

A. UKB

B. NHANES

C. NAHSIT

**Supplementary Figure 2. Age- and sex-specific normative values for metrics of lipid metabolism-related biomarkers in UKB**

**Supplementary Figure 3. Age- specific percentiles for muscle health metrics among women**

A. UKB

B. NHANES

C. NAHSIT

**Supplementary Figure 4. Age- specific percentiles for muscle health metrics among men**

A. UKB

B. NHANES

C. NAHSIT

**Supplementary Figure 5. Age- and sex-specific normative values for adiposity measures among women**

A. UKB

B. NHANES

C. NAHSIT

**Supplementary Figure 6. Age- and sex-specific normative values for adiposity measures among men**

A. UKB

B. NHANES

C. NAHSIT

**Supplementary Figure 7. Age- specific percentiles for biochemistry measures among women**

A. UKB

B. NHANES

C. NAHSIT

**Supplementary Figure 8. Age- specific percentiles for biochemistry measures among men**

A. UKB

B. NHANES

C. NAHSIT
